# Organic and Biogenic Nanocarriers as Bio-Friendly Systems for Bioactive Compounds’ Delivery: State-of-the Art and Challenges

**DOI:** 10.3390/ma16247550

**Published:** 2023-12-07

**Authors:** Sanja M. Petrovic, Marcela-Elisabeta Barbinta-Patrascu

**Affiliations:** 1 Department of Chemical Technologies, Faculty of Technology, University of Nis, Bulevar Oslobodjenja 124, 1600 Leskovac, Serbia; milenkovic_sanja@yahoo.com; 2 Department of Electricity, Solid-State Physics and Biophysics, Faculty of Physics, University of Bucharest, 405 Atomistilor Street, P.O. Box MG-11, 077125 Măgurele, Romania

**Keywords:** nanocarriers, bioactives, “green” technologies, biologically derived nanocarriers, biomimetics, bio-inspiration, bionic

## Abstract

“Green” strategies to build up novel organic nanocarriers with bioperformance are modern trends in nanotechnology. In this way, the valorization of bio-wastes and the use of living systems to develop multifunctional organic and biogenic nanocarriers (OBNs) have revolutionized the nanotechnological and biomedical fields. This paper is a comprehensive review related to OBNs for bioactives’ delivery, providing an overview of the reports on the past two decades. In the first part, several classes of bioactive compounds and their therapeutic role are briefly presented. A broad section is dedicated to the main categories of organic and biogenic nanocarriers. The major challenges regarding the eco-design and the fate of OBNs are suggested to overcome some toxicity-related drawbacks. Future directions and opportunities, and finding “green” solutions for solving the problems related to nanocarriers, are outlined in the final of this paper. We believe that through this review, we will capture the attention of the readers and will open new perspectives for new solutions/ideas for the discovery of more efficient and “green” ways in developing novel bioperformant nanocarriers for transporting bioactive agents.

## 1. Introduction

**Figure 1:**
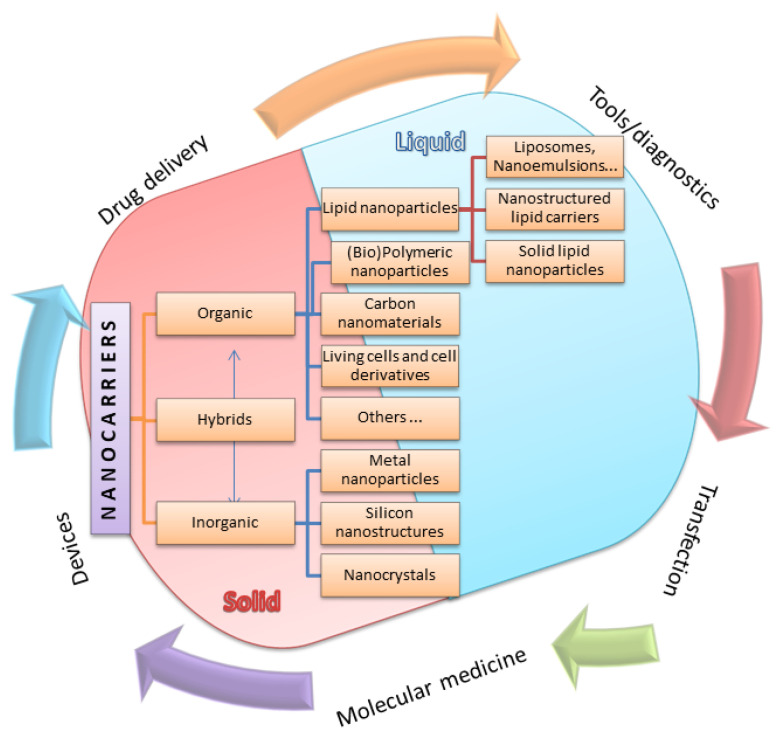
Different types of nanocarriers for bioactive compounds’ delivery.

Nanotechnology has made a significant contribution to the development of nanomaterials and nano-delivery systems with wide use in cosmetics, pharmacy, medicine, textiles, the food industry, engineering, materials science, chemistry, (bio)physics, and so many other areas. Particles where at least one dimension is smaller than 100 nanometers can be considered nanoparticles (NPs). In that broad world of nanomaterials and nanotechnology, the nanocarriers (NCs) are the nanoparticles (NPs) that allow the encapsulation of bioactive compounds (BCs) in the inner or in the outer regions of their structures (Figure 1). NCs improve the bioactivity of BCs and protect the encapsulated BCs against degradation produced by temperature, enzymes, and pH.

We often ask ourselves the question: *Why “nano”?* The answer is: In contrast to the objects at the macroscopic scale, the same materials/objects at the nano-scale have unique properties (such as interesting optical properties, high catalytic activity, bioperformances), due to their large area/volume ratio.

Nano-delivery systems or NCs for drug delivery (see Figure 1) must possess the following bio-physico-chemical features: (1) chemical inertness and biological stability under physiological conditions; (2) non-toxicity, non-immunogenicity, and biocompatibility; (3) good capacity of the encapsulation of active principles; (4) controllable retention time; (5) sustained release of active principles in final products; (6) specific function at the pathogenic site with a minimal impact on healthy tissues; (7) scalability; and (8) low-cost [1,2,3]. The pharmacokinetic properties of incorporated active principles including biological half-life and bioavailability (or bioaccessibility) are also improved resulting in better clinical and functional efficacy *in vivo*. The most common NCs, such as liposomes, micelles, nanoemulsions, polymeric NPs, and others have a broad spectrum of useful properties [1,2] such as to be small in size, biodegradable or to be easily eliminated from the body, to have a good drug binding capacity, enough space to maintain a high drug concentration, to keep the drug in an inactive form until it arrives at the target. In short, the requirements that the nanocarrier must fulfill are “Retain, Evade, Target, and Release”. 

Nanocarriers’ classification can be performed in several ways. One way is based on the aggregation state: solid (solid lipid nanoparticles (SLN), nanocrystals, nanospheres, polymeric nanoparticles) and liquid (emulsions, liposomes, nanopolymersomes) carriers [4]. The other is based on the form: (1) vesicular (liposomes, niosomes, virosomes, bilosomes, etc.), (2) particulate (poly(lactic acid) (PLA), functionalized carbon nanotubes (fCNTs), calcium phosphate nanoparticles), and (3) miscellaneous (dendrimers, bacterial carriers, polymeric micelles, etc.) [5]. The classification can also be made in regard to the organic and inorganic structures, and their hybrids, as shown in the Figure 1 [6,7,8]. The physicochemical properties of NCs can be tuned by altering their dimensions (small or large sizes), shapes (sphere, rod, hyperbranched, multilamellar, or multilayered structures), and surface properties as well (functional groups, surface charge, coating processes, or attachment of targeting moieties) [9]. 

The performances of NCs compared to other carriers are mostly due to their small size. For example, the passage of NCs loaded with active bio-drugs through permeable blood tumor vessels is more likely, as well as reducing the systemic toxicity and increasing efficacy of these drugs compared to conventional pharmaceutical forms of the same medicinal drug. 

This paper is a comprehensive review related to organic and biogenic nanocarriers (OBNs) for transporting bioactive compounds, providing an overview of the studies on the past two decades, from the WOS database. OBNs are nanostructures obtained from organic molecules (natural or synthetic), living systems (e.g., living cells), or bio-derived structures (e.g., hair, spider silk, biomembranes, cell derivatives, etc.). Between the (bio)organic nanoformulations carrying bioactive compounds, it could be mentioned the main types of nanocarriers (NCs): (1) polymeric NCs, (2) carbon-based nanomaterials, (3) biological NCs (based on lipids, biopolymers, living cells, and cell-derivatives), and (4) hybrid NCs. 

The fate of OBNs in the body, and their safety and toxicity, challenges and opportunities, limitations, and future strategies are discussed in dedicated sections. 

Taking into account the environmental problems that the world is facing today, it is imperative to use “green” technologies for OBNs development. In the last decade, the term “green” is more and more often used (e.g., green product, green technologies, green strategy, green method, green design, etc.), but what does it mean for something to be “green”? Nowak stated that the term “green” (product, strategy, technology, process, method, etc.) is something that does not cause any destructive impact on the environment and humans [10]; but, why green? Because the plants are green, and by adopting eco-friendly approaches, we keep the plants green colored, in a clean and healthy environment. Considering these, a “green” education is of real interest today. Among the synonyms of the term “green”, we mention the following: “eco-friendly”, “environmentally friendly”, “ecological”, “sustainable”, or “clean”. 

This review also brings the idea of adopting a “green” strategy by recycling natural residues (bio-waste, i.e., residues derived from living systems such as plants, animals, cells, etc.) for the production of bioactive nanocarriers (OBNs), for the following reasons:

(1) *Economic*: recycling raw materials greatly reduces production costs.
(2) *Ecological*: bio-waste is eco-friendly.
(3) *Biological*: bio-residues are bio-friendly, i.e., compatible with living organisms and are biodegradable and exhibit various bio-activities.

Moreover, the importance of valorizing vegetable waste is highlighted, since plants are found in abundance in nature, are ecological, and contain many ingredients that possess interesting bioactivities.

A recent review [11] pointed out that considerable research interest has been paid in recent years to phytochemicals encapsulated or conjugated with NCs for BCs’ delivery to the specific sites. On the other hand, phyto-ingredients are valuable bioactive compounds with antioxidant, antimicrobial, anti-inflammatory, antiproliferative properties. Future investigations can help in finding structural changes in NCs during digestion and absorption, and the impact on the phytochemicals’ metabolism [11].

This paper ends with the conclusions and future directions, highlighting the idea of valorizing natural waste in order to obtain performant organic and biogenic bioactive nanocarriers with huge potential for applicability in the biomedicine, nanotechnology, and food fields. 

Some important aspects regarding bioactive compounds are detailed in the following section.

## 2. Bioactive Compounds

### 2.1. General Aspects

Compounds that express biological activity can be considered *bioactive compounds* [12,13,14]. They are usually present in small quantities in fruits, vegetables, and whole grains. Unlike essential fats, carbohydrates, proteins, vitamins, and minerals, they are not essential for life and the body can function properly without them. These compounds have numerous positive effects like antioxidant, antidiabetic, antitumor, antibacterial, anti-inflammatory, etc. Beyond the basic nutritional value, BCs influence the metabolic processes by expressing antioxidant effects, inhibition of receptor activities, inhibition or activation of enzymes, and induction and inhibition of gene expression [15,16]. 

Antioxidants (vitamins, phyto-compounds, etc.), polyunsaturated fatty acids, probiotics, and proteins are common bioactives [4]. 

The plant kingdom is a rich source of BCs (known as *phytochemicals*) which have various biological activities (including antimicrobial, antioxidant, anti-inflammatory, or anticancer action). The phyto-BCs are valuable molecules with applications in the pharmaceutical, cosmetic, and food industries. In the last decade, special attention has been given to phyto-derived compounds. Such phyto-bioactives are phenolic compounds (e.g., phenolic acids, flavonoids, tannins, etc.), vitamins (e.g., ascorbic acid), carotenoids, phytosterols, and sulfur- (e.g., allicin, alliin, etc.) and nitrogen-containing compounds (e.g., alkaloids) [17]. These compounds have two major disadvantages: low solubility and instability in storage conditions or gastrointestinal conditions. These shortcomings were removed by the development of nanosystems capable of encapsulating BCs. There are clinically approved NCs for drugs, such as liposomes, albumin nanoparticles, dendrimers, polymeric and metal nanoparticles, and molecular targeted nanoparticles. 

A valuable bioactive phyto-compound with multi-faced pharmacological and biological activities (such as anticancer, antioxidant, antibacterial, anti-inflammatory, wound healing, and anticoagulant properties) is curcumin (CUR), a natural polyphenol found in the turmeric (*Curcuma longa*) rhizomes [18]. However, its biomedical application is limited by its hydrophobicity and fast degradation at a physiological pH. Thus, various nanoformulations have been developed for the protection of CUR [19], such as SLN, NLC, liposomes, and polymeric nanoparticles. Some herbal medicines such as nanoformulations that include CUR, resveratrol (RSV), and quercetin (Que) are used as anticancer agents against ovarian cancer (OC). OC is a silent killer because it does not make manifestations in the first stages and it is resistant to most of the known therapies. The nano-based formulations (e.g., liposomes, nanoparticles, etc.) of these phyto-compounds increase the solubility, bioavailability, and stability of BCs, which improve the mechanism of action against OC [20]. Resveratrol (RSV), derived from red grapes, peanuts, or berries, is a proven polyphenolic anticancer agent. Its nanoformulations, such as liposomes, polymeric nanoparticles, solid lipid nanoparticles, and lipospheres, improve RSV properties [21]. 

Another anticancer phyto-bioactive is honokiol (HNK)—a phytochemical derived from *Magnolia* usually used in Asian traditional medicine, due to its antioxidative, antiangiogenic, anti-inflammatory, and anticancer activities [22].

One of the representative chemotherapeutic antitumor drugs extracted from plants is paclitaxel (PTX) which is used for the treatment of breast, ovarian, non-small cell lung (NSCL), and other cancers. However, to enhance anticancer activity, several nanodrug delivery systems have been developed; for example, liposomes and albumin nanoparticles [23]. 

Naringenin, an anticancer phytochemical derived from fruits, tomatoes, and cherries, is less effective because of its hydrophobic nature when it is not incorporated in NCs. As a nanosystem, incorporated in nanostructured lipid carriers (NLCs), liposomes, solid lipid nanoparticles (SLNs), its therapeutic potential in the treatment of liver diseases, ocular disorders, skin, diabetic, and inflammatory diseases is improved [24].

Investigations in the past few years were carried out around the theme of glycyrrhetinic acid, because of its remarkable biological activity, natural sweetness, and good biocompatibility. Glycyrrhetinic acid has been incorporated in gels, micelles, and lipid nanoparticles, providing better bioactivities for functional applications [25]. 

Plant bioactives have been used in cosmetics as well, but their limitations are the poor penetration and stability of BCs. Nanosizing of phytocompounds can help reduce these limitations. *Aloe vera*, resveratrol, curcumin, vitamins C and E, quercetin, genistein and green tea catechins, gallic acid, epicatechins, hydroxybenzoic and cinnamic acids, luteolin, alpha- and beta-carotene, complex polysaccharides, and fatty acids, incorporated into nano-delivery systems, were the most commonly used nano-delivery agents in cosmetics [26]. 

The phyto-derived bioactives with use in the pharmaceutical, food and chemical fields, were isolated by appropriate and standard methods from plant materials by conventional methods depending on the nature of the plant matrix, chemistry of bioactive compounds, and scientific expertise [27]. The usually used techniques are Soxhlet extraction, maceration, and hydrodistillation, or the advanced “green” technology way by supercritical extraction [28,29,30,31]. 

However, natural bioactive compounds are chemically unstable and susceptible to oxidative degradation, particularly when exposed to oxygen, enzymes [32], light [33], moisture, and heat [34]. So, the incorporation of BCs into NCs is needed, in order to increase their bioavailability and their biological action. 

BCs can be inserted into carriers alone or together with other active compounds, to improve their bioactivities. Thus, the plant extracts usually contain a variety of bioactive molecules with synergistic action that are beneficial from a pharmacological point of view. Crude extract or fractions instead of a single isolated compound is interestingly chosen because of the possible synergistic effect of the extract compounds or multi-targeting effect [16]. However, sometimes it is hard to clarify and explain how those compounds are arranged within the carrier and how they interact with each other. 

The option of which carrier to choose depends mostly on the physico-chemical properties of a bioactive component. Whether a bioactive component is alone or accompanied by others in the extract, the localization of it could be expected as: (a) entrapped in the carrier, (b) onto the carrier surface, or (c) solubilized in the external phase [8]. The aqueous extracts (water is used as the extraction solvent), alcoholic extract (ethanol is used as the extraction solvent), hydroalcoholic extracts, and essential oils can be obtained from a plant material and inserted in the appropriate nanocarrier depending on its polarity. Extracts prepared with solvents with a lower dielectric constant like ethyl ether and dichloromethane are also common and used for the extraction of BCs as lipids, essential oils, waxes, and alkaloids in the free base form [8]. 

In Table 1, some phyto-derived bioactive compounds and the nanocarriers transporting them are mentioned.

### 2.2. Different Types of Bioactive Compounds and Their Relevance to OBNs

OBNs often incorporate various bioactive compounds depending on the intended application and the desired therapeutic outcome. These bioactive compounds can be derived from natural sources or synthesized for specific purposes. Bioactive components can be the main active components incorporated in the nanocarrier (like carotene incorporated in liposomes) or as the main building blocks thereof (liposomes made of phospholipids). 

The main types of bioactive compounds commonly used for the development of organic and biogenic nanocarriers are the following:

- **Lipids,** such as phospholipids and fatty acids, are fundamental components of lipid-based nanocarriers like liposomes and lipid nanoparticles [46,47]. They contribute to the formation of lipid bilayers and influence the stability and release characteristics of the nanocarriers.
- **Proteins,** including albumin, gelatin, and silk fibroin, are used to construct protein-based nanocarriers [48]. They provide biocompatibility, structural stability, and can be modified for targeted drug delivery.
- **Polysaccharides**, like chitosan, alginate, and hyaluronic acid, are frequently employed in the development of nanocarriers [49,50]. They offer biocompatibility, controlled release, and mucoadhesive properties.
- **Nucleic Acids,** including DNA and RNA, are used for the development of nanocarriers for gene delivery [51,52]. They can be encapsulated or conjugated to the nanocarrier surface for therapeutic applications.
- **Antibodies and Peptides** can be incorporated into nanocarriers for targeted drug delivery [53]. They enhance specificity by recognizing and binding to specific cell receptors.
- **Enzymes**, such as catalase or glucose oxidase, can be used to modify nanocarriers for targeted drug release or for responsive drug delivery systems [54].
- **Flavonoids and Polyphenols**, extracted from plants, are used as bioactive compounds for their antioxidant properties. They can contribute to the stability and bioactivity of nanocarriers [55].
- **Essential oils** derived from plants contain bioactive compounds with antimicrobial properties. They can be incorporated into nanocarriers for applications in antimicrobial drug delivery [56].
- **Plant extracts** contain an enormous variety of bioactive molecules which are valuable from a pharmacological point of view (e.g., essential oils, phenolic compounds, vitamins, alkaloids, etc.) [8].
- **Natural pigments (Bio-pigments)** originated from microbial, vegetal, or animal sources such as chlorophylls, carotenoids, anthocyanins, and melanin have applications in the food sector [57], or in the biomedical field [58], due to the antioxidant, anti-inflammatory, antimicrobial, radioprotective, and gastrointestinal benefits.
- **Vitamins** (such as vitamins A, B-complex, C, D, E, etc.) provide health benefits (e.g., antioxidant activity, modulation of the inflammatory response, and osteoporosis prevention, etc.) [59].

It can be mentioned that the use of plant-derived bioactive components not only contributes to sustainable nanoparticle synthesis but also extends their application to the development of potent drugs for combating a wide range of medical conditions. 

The main types of organic nanocarriers for the delivery of bioactives will be detailed in the following sections. 

## 3. Polymeric Organic Nanocarriers of Bioactives

Polymeric nanoparticles include various natural or biocompatible synthetic polymers. They can be divided into nanospheres which form a continuous polymer matrix and nanocapsules consisting of a polymer layer that closes a cavity filled with fluid. In general, polymer nanoparticles are very stable due to their rigid matrix [60]. Hydrophilic vesicles of 122 nm size, composed of poly(caprolactone)–poly(ethylene glycol)–poly(caprolactone) copolymers could penetrate the epidermis of human skin [61], and can be used in cosmetics and pharmaceutics. Polymeric materials such as polylactide (PLA), polyglycolide (PGA), copolymer poly(lactide-co-glycolide) (PLGA), poly(DL-lactide-co-glycolide) (DLPLG), and poly(ε-caprolactone) are materials approved by the World Health Organization as well as the US Food and Drug Administration (FDA) as materials that can be used in medicine and pharmacy. By homogenization of the aqueous and organic phases, various drugs (ascorbic acid, folic acid, proteins, etc.) were encapsulated in the polymer matrix and the degradation process was examined *in vitro* in different degradation media. Polymer nanoparticles have high biocompatibility and no toxic and thrombogenic effects in the body, which make them good candidates for drug carriers. Moreover, good water solubility and backbone stability are important factors contributing to the effectiveness of polymer nanoparticles as delivery systems for cancer immunotherapy using bioactive agents, and attenuating the immune side effects of cancer vaccines [62]. Polymeric nanoparticles as well as polymeric complex nanoparticles (e.g., cyclodextrins, nano-caseins, nanocrystals, and nano-spray dried particles) are some of the most effective carriers that can be used for the protection and delivery of phenolic bioactive compounds [63]. The biopolymer-based nanocarriers will be detailed in Section 5.2.

Many examples of polymeric nanocarriers with bioactive compounds are currently found in the pharmacy, medicine, cosmetics, textiles, foods, etc., and some of them are listed in Table 2. 

Polymeric nanomicelles are self-assembling structures in the range of 10 to 100 nm that are formed spontaneously above a critical micellar concentration. They are suitable for hydrophobic drugs incorporation in the hydrophobic core in the final liquid formulations. For example, nanomicelles prepared using polyoxyl 40 stearate, polysor-bate 80, D-alpha-tocopheryl polyethylene glycol succinate, octoxynol-40, and hydrogenated castor oil-40 have shown improved drug retention on the retina and choroid as well as negligible cytotoxicity [76]. In order to improve CUR solubility, chemical stability, and *in vitro* cellular uptake, CUR nanomicelles were prepared using polyvinyl caprolactam–polyvinyl acetate–polyethylene glycol for ocular inflammation treatment. The designed nanomicelles showed improved *in vivo* corneal permeation compared to the free CUR solution [77,78]. 

Novel polymer nanoparticles structures that consist of three distinct architectural regions, the core, and layers of branched repeat units emerging from the core, are known as *dendrimers*. They are able to carry both hydrophilic and hydrophobic active compounds [79]. The most common are poly(amidoamine)- PAMAM dendrimers, but they can be made of other polymers as well [80]. The modifiable branches of dendrimers enable them to selectively absorb a diverse spectrum of BCs, such as CUR, carotenoids, many phenolic compounds, etc., and to protect their contents from rough conditions, such as pH variations, enzymatic degradation, etc., which are rarely observed in the case of other NCs [81]. 

## 4. Drug Delivery Systems Based on Carbon Nanomaterials

Carbon-based nanomaterials (CBNs) are all nanomaterials composed of carbon atoms, and they can have different shapes, such as planar, tubular, spherical, and horn-shaped [82].

CBNs including carbon nanotubes (CNTs), fullerenes, carbon nano-onions (CNOs), carbon quantum dots (CQDs), and nanodiamonds (NDs) have attracted great attention in the biomedical field, especially as drug carriers due to their chemical, thermal, electrical, structural, mechanical, and optical properties and also due to their bioactivities (antioxidant, antimicrobial, and antitumoral properties), and structural diversity [83].

The CBNs functionalization with bio-based materials decreases their toxicity, and enhances biocompatibility. On the other hand, after fulfilling their function, especially in nanodelivery applications, CBNs have to be biodegraded in cells. The biological degradability of CBNs is still a challenge. A recent review [84] highlighted the role of the innate immune system in the enzymatic digestion of CBNs, and also the role of mammalian peroxidases, such as neutrophil myeloperoxidase (MPO), eosinophil peroxidase (EPO), and lactoperoxidase, in degradation of carboxylated single-walled carbon nanotubes (SWCNTs). 

The association of CBNs with phyto-bioactive compounds results in the enhancement of the bioactivities as compared to CBN or bioactive alone. In addition, the phyto-functionalized CBNs have reduced toxicity and enhanced biocompatibility, being potential candidates for biomedical applications. In this regard, de Carvalho Lima et al. suggested in their review [82] the exploration of the possibilities of using carbon-based nanomaterials combined with plant materials such as *Aloe vera*, as an antineoplastic agent in the prevention and treatment of melanoma. 

In this section, some CBNs (CNTs, fullerenes, CNOs, CQDs, and NDs) as nanocarrier systems will be further presented.

### 4.1. Carbon Nanotubes (CNTs)

Carbon nanotubes (CNTs) as biocompatible and supportive substrates for delivery systems are a newer generation of materials that have potential in novel applications in nanomedicine and pharmacy. Carbon nanotubes (CNTs) are an allotropic form of carbon, consisting of graphene sheets rolled into cylindrical (tubular) shapes. Graphene consists of a planar (two-dimensional) arrangement of carbon atoms (C–sp^2^) arranged in a regular hexagonal lattice, resembling a honeycomb structure that gives CNTs unusual properties: high mechanical strength (due to C–C sp^2^ bonds, one of the strongest in nature), flexibility without breaking or damage, high elasticity, high resistance to breaking, good electrical conductivity, chemical stability, biological activities (antioxidant, antimicrobial, and antiproliferative properties) [85], being considered today, the most attractive nanomaterials. CNTs usually have a diameter (d) of only a few nanometers, and the length (L) is of the order of micrometers. Therefore, the L/d ratio is very high, so that CNTs appear pseudo-one-dimensional (1D), looking like microsyringes—the shape that allows them easy penetration through cell membranes—making them ideal candidates in medical applications (e.g., in the development of drug delivery systems (DDS)). Depending on the number of graphene sheets (walls), CNTs are classified as: (i) single-walled carbon nanotubes (SWCNTs); (ii) double-walled carbon nanotubes (DWCNTs); (iii) multi-walled carbon nanotubes (MWCNTs). 

The important concerns of CNTs are their toxicity, biopersistence, and biocompatibility. As stated by Huang [86], the main factors responsible for the toxicity of CNTs are the following: geometrical parameters (length, diameter, surface area, structure defects), surface modification (e.g., functionalization), tendency for agglomeration, and residuals from the fabrication process. Functionalized CNTs exhibit minimal toxicity and are less immunogenic; [87,88] stated that the dispersion or functionalization of SWCNTs minimizes their toxicity to be applied in the biomedical field. For more information regarding the biocompatibility and toxicity of CNTs, the readers are suggested to go through the review of Sireesha et al. [89]. 

Regarding the CNTs bio-applications, studies have shown that cells trigger cascade reactions to resist CNTs-induced toxicity [85]. After fulfilling their role, CNTs must be degraded by cells (termed as *cellular degradation*). The biodurability or biopersistence of CNTs is then closely related to their toxicity. In this regard, Sireesha et al. [89] highlighted some aspects regarding CNT biodegradation. It has been found that functionalized SWCNTs which were dispersed in phagolysosomal simulant fluid were degraded inside cells, such as neutrophils and macrophages, by the action of myeloperoxidase. The authors pointed out that MWCNTs also degrade much slower than SWCNTs due to their concentric graphene sheets.

Compared to other nanocarriers, CNTs can be easily modified by the conjugation of bioactive compounds and ligands for targeting [90]. Many molecules, ions, or metals can be inserted. Fullerenes, porphyrins, and metals have indeed been included in the internal space of CNT, mostly due to hydrophobic interactions.

In their review [26], Ganesan and Choi pointed out that CNTs alone act as an antioxidant at 100 nm in size, and they are effective in phytocompound-based delivery systems for bioapplications. As known, many phyto-constituents possess antioxidant, anti-inflammatory, and/or antitumor properties; therefore, their conjugation with CNTs results in the enhancement of these bioactivities, and also in reducing CNTs toxicity. Thus, Ding et al. [91] constructed a bionic mesh structure coating for tissue-engineered blood vessels (TEBVs) based on single-walled carboxylic acid functionalized carbon nanotubes loaded with resveratrol (CNT-RSV). CNT-RSV presented reduced cytotoxicity and anti-inflammatory activity due to the resveratrol. Moreover, CNT-RSV revealed a better antioxidant activity compared with RSV and CNT alone.

Li et al. [92] developed a novel delivery system for CUR using functionalized SWCNTs with phosphatidylcholine and polyvinylpyrrolidone (SWCNT-CUR). SWCNT-CUR improved CUR bioaccessibility resulting in an enhanced anticancer activity by the adequate CUR delivery to tumors. 

The research group of Saheeda [93] developed a smart nanocomposite of MWCNTs with polypyrrole (PPy) via an eco-friendly route by using lemon fruit extract. This nanocomposite presented enhanced biocompatibility and low toxicity, and enhanced curcumin encapsulation of about 89%, and also exhibited pH dependent and sustained drug release over a prolonged period, offering great promise in drug delivery applications.

CNTs’ biofunctionalization with artificial cell membranes is an effective “green” strategy to increase CNTs biocompatibility and their bioactivities. In this regard, SWCNTs were biodispersed in chlorophyll a-loaded biomimetic membranes to build up multifunctional quercetin (Que) nanocarriers [94] with high antibacterial properties against *Staphylococcus aureus*, and enhanced antioxidant activity (85%, estimated by the chemiluminescence technique). 

### 4.2. Fullerenes

Fullerenes or buckminsterfullerenes can be considered another allotropic form of carbon. They contain a high and even number of carbon atoms (e.g., C60, C70, C72, C74, C76, C78, C84, etc.) with a typical cage arrangement in “geodesic dome”-type structures, with sp^2^ carbon atoms. Fullerenes are promising candidates for biomedical applications, their cytotoxicity being lesser than that of CNTs [84]. 

C60, the smallest stable fullerene, is known to be a powerful antioxidant and anti-aging agent. In a study, Baati et al. showed that repeated oral administration of C60 suspension in olive oil prolonged the lifespan of rats [95].

### 4.3. Carbon Nano-Onions (CNOs)

Multi-layer fullerenes, known as carbon nano-onions (CNOs), which were first discovered in 1992, possess interesting physico-chemical properties and biocompatibility, enabling their use in biomedical applications. CNOs have been used as nanovehicles for the delivery of glycopeptides and proteins [96], and flavonoids such as Que [97]. 

### 4.4. Carbon Nanodots

Another class of carbon nanoparticles is carbon quantum dots (CQDs), also known as carbon nanodots, which are less than 10 nm in size [98]. CQDs were discovered accidentally by Xu et al. [99] while attempting to purify SWCNTs. CQDs possess unique features including low toxicity, outstanding biocompatibility, good water solubility, bactericidal potential, chemical inertness, and environmentally friendliness [98]. CQDs “green” synthesized from biomolecules (e.g., folic acid, vitamins, carbohydrates, amino acids, glutathione, proteins, nucleic acids) and plants (e.g., basil, green tea, bamboo, wood) [100,101] are also known as *Biodots* and they have great potential in biomedical applications such as cell-imaging and sensing, fluorescent ink, and drug delivery. 

CQDs are classified as zero-dimensional materials. Their small size enables the facile penetration of CQDs into cell membranes, thus having great potential for bio-applications, especially as drug nanotransporters [102]. Carbon dot-based nanoplatforms were used in the biomedical field for carrying proteins [103], Que [104], melatonin [105], or chlorophyll (Chl) [106] with high therapeutic potential in cancer treatments. 

### 4.5. Nanodiamonds (NDs)

Another type of CBN explored as a novel drug delivery nanomaterial are nanodiamonds (NDs) which are carbon nanoparticles with a size of 2~8 nm [107]. NDs produced by a detonation reaction are more suitable for drug delivery because they have a large number of organic functional groups (carboxylates, amines, esters, ethers, lactones, etc.) facilitating functionalization with a wide range of bioactive molecules [21]. These “magic bullets”, NDs, were used to deliver RSV [21] for inducing apoptosis in tumor cells. Another research group improved the oral delivery efficiency of the hydrophobic bioactive molecule CUR [107] by the non-covalent or covalent conjugation of NDs with d-alpha-tocopheryl polyethylene glycol 1000 succinate (TPGS), and then loaded with CUR (CUR@NDs-COOH/TPGS or CUR@NDs-TPGS). 

## 5. Biogenic Nanocarriers

Biogenic nanocarriers (BNCs) are delivery systems composed of natural/biological compounds, bio-entities, or living systems like entire cells or parts of cells or cell wastes. They impart excellent bioactivities including biocompatibility, bioavailability, biodegradability, and low/no toxicity. BNCs improve the biological properties of the bioactives when inserted in them. 

### 5.1. Lipid-Based Nanocarriers for Bioactive Compounds

Lipid-based nanocarriers (LNCs) are the most attractive systems to transport bioactives, due to their unique features: biodegradability, biocompatibility, and entrapment capacity of both hydrophilic and hydrophobic therapeutical agents. The main categories of LNCs used for the delivery of bioactive compounds are liposomes, ufasomes, phytosomes, terpesomes, aspasomes, niosomes, nanoemulsions, emulsomes, bilosomes, solid lipid nanoparticles, and nanostructured lipid carriers. Examples of some nanolipid carriers with BCs used in different industries are given in Table 3.

The vesicular LNCs are the most widely used in drug delivery, since they overcome many problems related to the carried drug such as insolubility, biodegradation, and difficulty in penetrating the skin and biological barriers [127]. The structure of vesicular-type DDS allows the encapsulation of both hydrophilic and hydrophobic active molecules. Furthermore, the amphiphilic nature of lipids is responsible for the biomimetic character allowing the affinity of LNCs toward biological species, especially toward the biomembranes [128]. 

An important application of nanovesicular drug nanocarriers is the treatment of autoimmune diseases. Autoimmune diseases cause the body’s response, leading to disorders like arthritis, neurodegenerative diseases, etc. With liposomes, transferosomes, ethosomes, ufasomes, and other drug carriers, the therapeutic efficiency of autoimmune disease drugs have been improved [129]. 

Some examples of different **lipid nanovesicular carriers** used in DDS formulations will be briefly described further.

#### 5.1.1. Liposomes

The first and most successful type of delivery systems are vesicular nanoliposomes or nano lipid vesicles. 

Liposomes are self-assembled lipid vesicles composed of one or more phospholipid bilayers (membranes) separated by aqueous compartments. Since their discovery by Bangham and his collaborators in 1965, they have received great attention from the scientific world, being used in many fields, especially in the biomedical one, as carriers for both hydrophilic and hydrophobic compounds [130]. 

Liposomal membranes are very similar to natural cell membranes; therefore, liposomes are an example of bioinspiration and they have also been used as models of cell membranes. Despite their advantages (biocompatibility, biodegradability, high encapsulation efficiency, the ability to carry both hydrophilic and hydrophobic compounds, and transport ability through cell membranes), liposomes face many challenges such as poor physical and chemical stability, rapid elimination from blood circulation, low resistance to gastrointestinal environments, considerable loss of entrapped compounds, and lack of stimulus responsiveness. A solution to overcome these drawbacks is the coating with biopolymers (proteins, polysaccharides, etc.). These biopolymer–liposomes systems have been successfully applied as carriers for bioactive compounds, and have demonstrated superior properties over conventional liposomes in terms of improved bioavailability and therapeutic efficiency, high retention efficiency, and tolerance to environmental stresses [130]. 

The nano-sized liposomes, called *nanoliposomes* or *nanosomes*, could penetrate into small blood vessels via intravenous injection, and the encapsulated drugs can be easily transported and delivered to target cells [131]. For the first time, in 2020, Hsiao and collaborators [131] have employed nanosomes for the encapsulation of HNK, a hydrophobic phytochemical with antiangiogenic, neuroprotective, anticancer, and anti-inflammatory activities [22]. In order to increase the stability of the nanoliposomes and to moderate the fluidity of bilayers, cholesterol is used in a high concentration up to 1:1 or 2:1 (cholesterol to a phospholipid such as phosphatidylcholine). It does not by itself form bilayer structures, but it can be incorporated into phospholipid membranes. The amount of cholesterol depends on the bioactive compound properties, structure, and purpose of the obtained nanosystem [132]. For example, some investigations reported that liposomes with 40% or more cholesterol content could not be useful in gene and drug delivery applications [133,134].

Many articles and patents on nanoliposomes are published from 2002 [133] indicating the increased interest in the field of nanoliposome research. They have been also reported to be used as the delivery systems of enzymes, drugs, hormones, blood factors, antigens, diagnostic materials, vaccines, cosmetics, and foods, because of high encapsulation efficiency, long-term stability, ideal release properties, and a narrow size distribution. However, compared to liposomes, nanoliposomes provide more surface area and have the potential to increase solubility, enhance bioavailability, improve the controlled release, and enable precision targeting of the encapsulated BCs [133]. In addition, since nanoliposomes are made of phospholipids that are organic, they are good candidates for organic products with no toxicity to human health which is noticed for some NCs. The incorporation of BCs in liposomes is possible for nonpolar, polar, or amphiphilic compounds in different locations within the liposome structure [108,135]. The liposome structure itself is composed of phospholipids in which the polar heads are oriented towards the outer part of the vesicular structure and the phospholipid tails towards the inner part. Thus, oriented phospholipids form vesicles that can trap nonpolar substances within nonpolar phospholipid tails, or polar ones within internal vesicles or between the polar heads of phospholipids. For example, the localization of Chl, which has an amphiphilic character, along the nanoliposome of 100 nm size is such that the porphyrin structure as a polar part of that molecule is trapped between the polar heads of phospholipids, while the tail of the Chl structure as a nonpolar part of that molecule is located between the phospholipid tails of liposomes [136,137]. Once inserted, chlorophylls in liposomes have a better distribution in the artificial lipid bilayers, and are protected from numerous external stresses. Since bioactive Chl is very unstable [32,33,34,108,137,138,139,140], its use is limited, and such and similar systems as liposomes allow prolonged activity of Chl as well as reduced Chl degradation. This bioactive molecule—Chl—was used as a spectral sensor to detect the molecular events in the biomimetic membranes, including the effect of local anesthetics [141], oxidative stress studies [142], and monitoring the biohybrid formation [143]. 

Similar to this, the use of nanoliposomes to encapsulate and protect vitamins and other BCs has a number of positive aspects [144]. For example, liposoluble vitamin E mixes perfectly with the hydrophobic area of phosphatidylcholine [145]. Vitamin C encapsulated in liposomes retains 50% of its activity after 50 days in refrigerated storage, whereas a non-encapsulated vitamin loses its activity after 19 days. Liposomes also present an important protective effect over thermolabile vitamin C and show an antioxidant action after pasteurization [146]. Moreover, the encapsulation of vegetal extracts in liposomes resulted in an increase in their stability and their biological activities. Thus, impressive antioxidant activities were achieved when loading vegetal extracts of sage (*Salvia officinalis* L.) and mint (*Mentha piperita* L.) into nanoliposomes [147].

The methods for nanoliposomes’ preparation can be different depending on the character of a bioactive compound and of the nanoliposomes form: sonication technique, extrusion method, microfluidization, heating method, or the Mozafari method [108,133,148]. The development of nanoliposomes with bioactive peptides (carnosine, palmitoyl tripeptide-5, and acetyl hexapeptide-3) for improving anti-aging effects in human skin showed high encapsulation efficiency and loading capacity and the sustained release of bioactive peptides [149]. For nanoliposome systems, there are certain shortcomings such as low entrapment efficiency, instability, and a high cost of production especially in the scale-up level [111]. Liposome coating is a useful strategy to increase the stability of liposomes, and to improve the controlled/targeted release of active compounds [150] Thus, biopolymers applied in single, double, or multilayers for liposome coating conducted to biohybrid systems more efficiently for the protection and controlled delivery of bioactives (e.g., vitamins, carotenoids, peptides, phenolics, and other health-related compounds) [130].

Liposomes have also been used in the food industry for improving flavor and promoting antioxidant activity with the synergistic delivery of ascorbic acid and tocopherols in foods, but also to stabilize some minerals, such as iron in milk [151]. Furthermore, in respect to the industry of cheese, the liposomal entrapment of casein can protect the cheese from early hydrolysis during production [152]. Nanoliposomes are able to provide the protection and release of sensitive food-grade bioactive materials.

There is a lot of variation in the liposomes composition and structure resulting in various LNCs that classify into sphingosomes, marinosomes, ethosomes, transferosomes, niosomes, bilosomes, aquasomes, enzymosomes, virosomes, exosomes, etc. [118]. 

In the following, some examples of LNCs and their role in the transport of bioactive molecules/agents will be briefly presented.

#### 5.1.2. Ufasomes 

Ufasomes are unsaturated fatty acid liposomes made up of especially oleic and linoleic acids which are themselves bioactive compounds. Octanoic acid and docosahexaenoic acid were also used to prepare this kind of lipid nanocarrier. Ufasomes were proposed for the first time in 1973 by Gebicki and Hicks as “unsaturated fatty acid vesicles” [153]. Compared with liposomes, the ufasomes present a dynamic nature due to the presence of single-chain amphiphiles in their composition, and are able to improve the bioavailability of unsaturated fatty acids. These NCs are also able to deliver low-soluble bioactives such as oleuropein—a phenolic compound mainly present in olives and olive oil, with many bioactivities (e.g., antioxidant activity, antiviral power; hypoglycemic effects; anti-aging effects; anti-inflammatory properties; neuroprotector effect preventing hypoxia and ischemia; antitumoral activity). Cristiano et al. developed oleuropein-loaded ufasomes with enhanced antioxidant activity as compared to the free active substance [153]. 

#### 5.1.3. (Nano-)Phytosomes (Herbosomes)

(Nano-)Phytosomes are lipid nanocarriers that are made by mixing phosphatidylcholines (PC) with phytocompounds resulting in a complex with stronger bonds, offering advantages such as a better stability profile, high entrapment efficiency (EE%), and increased absorption. Such a nanocarrier system was prepared by Albash et al. [154]. They achieved novel nano-phytosomes loaded with bergamot essential oil (BEO) and combined them with spironolactone in order to use them against acne vulgaris. Cholesterol was incorporated into these vesicles to form more rigid and ordered membranes, thus reducing the BEO leakage.

#### 5.1.4. Terpesomes 

Terpesomes (TPs) are vesicles composed of terpenes and phospholipids. Terpenes like limonene, fenchone, and eugenol (EUG) are compounds derived from essential oils and are made up of several isoprene units, and possess both antimicrobial and penetration enhancer properties [154,155]. TPs are used as drug carriers to treat ocular diseases such as ocular candidiasis [156]. 

#### 5.1.5. Aspasomes 

Aspasomes are the newer antioxidant generation of liposomes; they are nano-vesicular antioxidant systems containing *ascorbyl palmitate* (a liposoluble vitamin C derivative), *cholesterol*, and *phospholipids* that have the ability to form stable vesicular bilayers [157]. The preparation, characterization, and applications of aspasomes were firstly reported in 2004 by Gopinath et al. [158]. Since then, aspasomes have been successfully used as nanocarriers for the dermal and transdermal delivery of various drugs and/or bioactives for the treatment of skin injuries (e.g., acne, melasma, psoriasis, fungal infections, skin cancer) [157,159,160,161,162], or for the management of rheumatoid disease [163] and muscle spasms [164]. Hatem et al. developed aspasomes loaded with melatonin, a bioactive antioxidant molecule, as a novel cosmeceutical for the clinical treatment of androgenic alopecia [165]. Aspasomes loaded with bioactives such as Que or several essential oils, namely tea tree and neem oils, displayed enhanced anti-inflammatory properties, proving to be successful in the treatment of acne vulgaris [159].

#### 5.1.6. Bilosomes

Bile salts inserted in liposomes, called bilosomes (BILs), possess a long residence time in the gastrointestinal tract (GIT) and permeability across the small intestine, being promising bio-nanoplatforms for the oral, intravenous, and topical drug delivery of active cargos [166,167]. Various BILs formulations were used as carriers of many bioactive molecules including silymarin (SYL) [168], berberine (BER) [169], CUR [170], and RSV [171], and have been applied for the management of the different diseases (e.g., skin damage, cancer, inflammations, Alzheimer’s disease, etc.). Moreover, BILs have been loaded with plant extracts such as cranberry extract (CBE) (with good hepatoprotective impact) [172], or *Bacopa monnieri* extract (BME) for memory enhancement [173].

#### 5.1.7. Quatsomes

Quatsomes (QS) are outstanding new lipid-based unilamellar nanovesicles composed of sterols and quaternary ammonium surfactants. In contrast to liposomes, QS are stable for several years and they are useful platforms for the site-specific delivery of hydrophilic and lipophilic molecules [174]. 

Interestingly, none of the individual components of a quatsome spontaneously aggregate into vesicular structures, given that the quaternary ammonium surfactant forms micelles in aqueous media, while the insoluble sterol species form crystals [175]. Ferrer-Tasies and co-workers studied the molecular origin and driving force of the synergy between both these molecular entities, governing the self-assembly of sterol/quaternary ammonium surfactant mixtures into exceptionally homogeneous bilayer vesicles [175]. 

Battista et al. [176] developed QS loaded with (+)-Usnic acid (UA), a bioactive substance produced by many lichens, that have antimicrobial, antiproliferative, and anti-inflammatory properties. These QS-UA formulations showed good antibacterial activity, being good candidates for the treatment of bacterial infections. 

#### 5.1.8. Niosomes

Niosomes are non-ionic surfactant-based vesicles often containing cholesterol or its derivatives as excipients, which increase the stability and influence the fluidity and permeability of niosomal membranes. They were developed for the first time in the 1970s, by the French personal care company L’Oreal [128]. 

Niosomes have been used in medical applications as nanocarriers for delivering novel drugs and bioactive agents both hydrophobic and hydrophilic in nature. The research group of Gunes [177] obtained and characterized niosomes made from Tween 60 (non-ionic surfactant) and a cholesterol mixture (1:1 mole ratio) containing the herbal extract of oleander (*Nerium oleander*) (ONs). These ONs vesicles were nano-sized and presented good physical stability for more than 50 days, as well as good antioxidant properties.

#### 5.1.9. Soysomes

Soysomes are a new class of biobased NCs, derived from soybean oil and sucrose. They were discovered by Chitemere et al. in 2018 [178], who prepared for the first time nanoparticles from methoxylated sucrose soyate polyol using a nanoprecipitation method. They observed that these self-assembled NPs were able to encapsulate and stabilize hydrophobic molecules, such as CUR, in the aqueous phase and release them in a controlled manner. This was an interesting idea to use soybean oil derivatives to develop valuable, highly biocompatible, and non-cytotoxic NCs for bioactive hydrophobic molecules. 

#### 5.1.10. Nanoemulsions

The emulsion is a dispersion of a liquid (droplets) in an immiscible liquid. The presence of an emulsifying agent is required for emulsion stabilization [179]. 

Emulsions of a nano-size are called *nanoemulsions*, and are involved in various applications. Nanoemulsions with a modified surface, by PEGylation or a hydrophilic, polyethoxylated surfactants coating, have an additional advantage because they can provide improved drug delivery to the specific sites. Nanoemulsions are used as carriers of bioactive compounds like Que, β-carotene, RSV, lutein, capsanthin, vitamin E, and CUR, because these systems are well known to enhance the kinetic, solubility, bioactivity, and physical stability [180]. In general, nanoemulsions are also known as mini-emulsions, ultrafine emulsions, or sub-micron emulsions, and they are generally described as oil-in-water emulsions with droplet sizes below 1000 nm, most commonly in the range of 100–500 nm [181,182], although a range of 50–200 nm is also reported. Depending on the droplet size, nanoemulsions can be transparent if the droplet size is below 100 nm, or milky if the droplet size is above 100 nm, and a bluish tint indicates the appearance of Rayleigh light scattering from small nanoemulsion droplets [183]. In general, nanoemulsions with a droplet size higher than 200 nm are preferred for food applications [181,184]. Thanks to the numerous advantages, the possibility of obtaining a small, homogeneous, and stable droplet size, and relatively simple scale-up, the high-pressure homogenization (HPH) technique is the method of choice for obtaining nanoemulsions, especially those based on lecithin. Such lecithin-based nanoemulsions with a bioactive component such as tocopherol, butylhydroxytoluene, ascorbic acid, sodium metabisulfite, cysteine, etc., are common parenteral preparations which, in addition to the mentioned antioxidants, most often contain vegetable oil as an oil nanoemulsion phase.

#### 5.1.11. Emulsomes

Another type of lipid-based nanocarrier with an internal solid fat core surrounded by a phospholipid multilayer are *emulsomes*. Ucisik et al. developed a DDS by encapsulating CUR in emulsomes, achieving the so-called CurcuEmulsomes [185]. These nanocarriers not only facilitated the CUR delivery to the human liver carcinoma cell HepG2 *in vitro*, but they also improved the CUR biological effect by controlled drug release inside the cell. 

#### 5.1.12. Solid Lipid Nanoparticles (SLNs) and Nanostructured Lipid Carriers (NLCs)

SLNs are composed of lipids that are solid at a physiological temperature, and are stabilized in an aqueous dispersion with the help of emulsifying agents [186,187], while NLCs are made up of a mixture of solid and liquid lipids. SLNs and NLCs are attractive nanocarriers derived from oil/water emulsions. These NCs show biocompatibility and are able to transport both lipophilic and hydrophilic drugs. 

Khatamian and collaborators developed SLNs loaded with myricetin, a flavonoid with anticancer properties. These SLNs were decorated with chitosan (CS) and active-targeted with folic acid (FA) [188]. The obtained myricetin-SLN-CS-FA exhibited high antioxidant activity and anticancer activity against human breast carcinoma MCF-7 cells.

NLCs are improved systems of SLNs, showing better stability and higher encapsulation efficiency for many bioactive compounds. Moreover, NLCs prevent the active substances’ expulsion from the lipid matrix during storage. Coc et al. prepared NLCs loaded with a hydrophilic compound, caffeic acid, and a hydrophobic one, linseed oil, which exhibited the enhancement of the antioxidant activity of both natural actives due to a synergic effect [189].

### 5.2. Biopolymer-Based Nanocarriers

Biological polymers such as peptides, proteins, polysaccharides, and nucleic acids are a good and effective alternative to synthetic polymers for the development of biocompatible and biodegradable nanovehicles for bioactives’ delivery. The main natural sources of biopolymers are plants (e.g., maize, soybean, wheat, rice, potatoes, banana, tapioca, corn, barley, etc.), animals (cattle are the most common animal sources), marine sources (corals, sponges, fish, lobster, and shrimp), micro-organisms (algae, fungus, and yeasts) [190], and agricultural and food industry wastes. 

#### 5.2.1. Protein and Peptide-Based Nanoformulations 

Proteins and peptides occurring in living organisms are both natural and polymeric, and have been normally found in the body, being successful candidates as drug nanocarriers. They are biodegradable and biocompatible, and do not release any harmful compounds when consumed orally and digested *in vivo* [191,192]. 

Protein and peptide-based nanoformulations have been registered with great progress in recent years, especially in cancer therapy, due to their greater specificity, bioactivity, penetrability through cellular membranes (especially for peptides), and low/no toxicity for healthy cells. Some drawbacks such as a short half-life in circulation and vulnerability to enzymatic degradation can be overcome through the nanoencapsulation of protein/peptide therapeutics that can preserve their structural integrity and can orientate to the target sites [193]. The preparation of protein nanoformulations and the corresponding encapsulation is a “green” process involving mild conditions without the use of toxic chemicals or organic solvents [194]. It must be pointed out that proteins and peptides can be used to design nanocarriers for transporting bioactives or they themselves can be encapsulated as bioactive agents in various NCs (e.g., liposomes, albumins, polymeric, or carbon-based NPs). Sorolla et al. stated in their review [193] that peptides, proteins, and nanotechnology provide a promising synergy for breast cancer therapy. Electrostatic interactions between the charged groups present in protein and in drugs facilitate the physical entrapment of encapsulated drugs [195]. The main proteins used in nanocarrier formulations include albumins, gelatin, gladin, legumin, zein, fibroins, casein, and ferritin. 

##### Proteins and Peptides as Bioactives

The main sources of bioactive proteins/polypeptides are milk, meat, eggs, cereals, fish, seaweeds, vegetables, and fungi. A recent review [196] highlights the impressive anti-tumor effects of bioactive proteins/polypeptides against breast cancer.

Milk is an important source of bioactive proteins and peptides. For example, casein, whey, and their peptides possess antimicrobial, anti-inflammatory, immunomodulatory, and cytomodulatory effects [197]. Other bioactive peptides derived from lactoferrin have antimicrobial, antioxidant, and anticarcinogenic properties.

Breast milk contains a multitude of bioactive proteins and peptides with immunomodulatory and antimicrobial activities, an important role in gut development and digestive function or as carriers for other nutrients. Therefore, breastfeeding is important for the healthy growth of infants and young children [198]. 

Camel milk is known for its unique proteins and bioactive peptides which exhibit antioxidant and antimicrobial properties, and angiotensin-converting enzyme (ACE) inhibitory activity. The protein pattern of camel milk is more alike to human milk than bovine milk [199].

Liu et al. pointed out the biological activities, including antioxidant, antimicrobial, anti-inflammatory, antidiabetic, angiotensin I-converting enzyme (ACE) inhibitory (antihypertensive), and iron-/calcium-binding activities, of bioactive peptides derived from egg proteins [200].

##### Proteins and Peptides as Nanocarriers for Bioactives

Protein-Based Nanocarriers possess many advantages for delivery applications such as biocompatibility, biodegradability, and the ability to functionalize with targeting ligands. They are safe materials and their preparation process occurs under mild conditions [201].

There are two types of protein-based NCs [201]:

(i) ***NCs based on animal proteins*** (e.g., albumin, gelatin, casein, whey, collagen, fibrinogen, silk, and elastin). They possess many advantages:
  - The presence of all essential amino acids in their composition;
  - Anionic proteins like albumins have low opsonization activity in the blood stream.
(ii) ***NCs based on plant proteins*** (e.g., zein, soy protein, gliadin, legumin, and vicilin)
  - Plant proteins show many advantages over animal proteins as follows: low cost, high availability, and simple purification steps.

(i) ***NCs based on animal proteins***

**Albumins** such as human serum albumin (HSA), bovine serum albumin (BSA), egg white albumin (ovalbumin, OVA), and rat serum albumin (RSA) have been used for different biomedical applications, including nanocarrier development, since they are biocompatible, biodegradable, noninflammatory, and nonimmunogenic. Albumin-based nanocarriers have the ability for the delivery of many bioactive molecules such as vitamins, carotenoids, phenolic compounds (quercetin, catechins, and chrysin), hormones, and various hydrophobic plasma agents [201,202,203]. 

HSA is mostly preferred due to its antioxidant properties, its less immunogenicity effects, and the ability of binding and transporting other molecules [201]. Moreover, HSA-based systems are nontoxic, biodegradable, non-immunogenic, and further favored in uptake in inflamed tumor tissues [195]. 

Another interesting aspect is related to the potential use of albumin complexes as DDS. In this regard, spectral and molecular docking experiments reported in a recent study [204] brought a significant contribution regarding the implementation as DDS of a complex between BSA and levothyroxine (LT4, the chemical equivalent of the thyroid hormone T4), BSA-LT4. The researchers found that the vitamins could influence the interaction between serum albumins and LT4. 

**Albusomes.** Proteins can be also used as a coating for various NPs, in order to increase the biofunctionality of the bioactive carried in these NPs. One such example is *albusomes*—albumin particles entrapping liposomes which are considered “green pharmaceutical vehicles” for the delivery of both hydrophilic and hydrophobic drugs [205]. Albusomes are more bioperformant than liposomes. Taguchi et al. highlighted that albusomes containing CUR are more spherical, more homogeneous in size, and present a considerable reduction in macrophage phagocytosis as compared to liposomal CUR [206]. 

**Human serum albumin (HSA NPs)**. HSA is an important nanocarrier of hydrophobic drugs. Thus, human serum albumin nanoparticles (HSA NPs) were used to deliver various BCs including CUR, PTX, docetaxel, methotrexate, all-trans-retinoic acid—a derivative of retinoic acid, known as vitamin A acid—and gambogic acid, with great potential in cancer therapy [38]. Zhang et al. developed HSA NPs encapsulating a disulfide bond bridged paclitaxel-pentadecanoic acid conjugate (HPTX NPs), via hydrophobic and electrostatic interactions [23]. The obtained HPTX NPs showed the advantage of reduced systemic toxicity compared to classical Taxol formulations, and exhibited good antitumor activity against four human cancer cell lines, MDA-MB-231, MCF-7, KB, and HeLa. Mohammad-Beigi et al. prepared HSA NPs coated with polyethylenimine (PEI) and loaded with gallic acid (GA), as an antioxidant and anti-Parkinson agent. PEI-HSA NPs proved to be good candidates for the efficient and safe delivery of GA to the brain [207].

**BSA nanoparticles (BSA NPs)** were successfully used to deliver salicylic acid, doxorubicin, PTX, rutin, CUR, β-carotene, gambogic acid, and piceatannol, for the treatment of cancer, cardiovascular, and inflammatory diseases [38].

**Fibrinogen Nanoparticles (FNPs).** Fibrinogen is a large plasma glycoprotein (with a molecular mass of 340 kDa), and is a key protein in the regulation of both thrombosis and homeostasis. Rejinold et al. prepared FNPs loaded with CUR [208] with great potential in applications as therapeutic agents for cancer treatment. CUR-FNPs were non-toxic to L929 (mouse fibroblast) healthy cells, but were toxic to PC3 (prostate) and MCF7 (breast) cancer cell lines. 

**Milk-protein nanocarriers.** Milk-derived NCs have been used as nanovehicles that deliver chemotherapeutic agents. Tavakoli et al. [191] recently reviewed various nanodrug delivery systems containing the milk proteins lactoglobulin, lactoferrin (Lf), and caseins, which were designed for the treatment of different cancer types. Interestingly, some of these proteins, such as lactoferrin, act as an antiproliferative agent. Some milk-protein nanocarriers will be further briefly described.

**Lactalbumin nanocarriers.** α-Lactalbumin (α-Lac) is a protein rich in essential amino acids (especially tryptophan, lysine, and cysteine), and it has antibacterial and antitumor properties, and also regulates lactose synthesis and milk secretion. The self-assembling of amphiphilic α-Lac peptides results in nanotubes (NTs) in the presence of calcium ions; these α-Lac NTs have been proven to be excellent NCs for hydrophobic bioactive compounds like carotenoids. Among many carotenoids, lycopene (LYC) has the strongest antioxidant activity, and its consumption can prevent cancer and cardiovascular diseases. Chang et al. developed α-Lac NTs loaded with LYC. LYC was inserted into the hydrophobic inner wall of NTs via hydrophobic interaction [209]. α-Lac NTs- LYC was successfully added to the dairy drink, showing an increased viscosity and a long-term stability, being a promising nanocarrier in protecting and delivering the hydrophobic bioactive compounds in the food system. 

**Casein.** β-Casein, a highly amphiphilic calcium-sensitive phosphoprotein, has been applied as a nanocarrier for hydrophobic bioactives such as luteolin (LUT) [42]. LUT encapsulated in beta-casein nanocarriers demonstrated higher antioxidant activity as compared to free LUT. Camel milk is similar to human milk, and contains many bioactive peptides with high antioxidant activity, antimicrobial activity, and ACE inhibitory activity. Moreover, camel milk-derived casein micelles are excellent NCs for bioactives [199].

**Lactoferrin (Lf).** Lactoferrin (Lf) is a cationic iron-binding glycoprotein, belonging to the transferrin (TF) family. Lf is found widely in human and mammalian milk, and possesses many biological activities including antioxidant, anti-inflammatory, antibacterial, antiviral, anticancer, and immuno-regulatory properties [210,211]. Lf is a valuable material that could be exploited both as an active therapeutic and drug nanocarrier [211]. A recent study [212] reported the preparation of self-assembled nanogels containing Lf and carboxy methyl cellulose (CMC) for the combined delivery of the hydrophilic antineoplastic agent pemetrexed (Pm) and the herbal polyphenol HNK. These delivery nanosystems showed a sustained release profile for both drugs (Pm and HNK), and demonstrated superior *in vitro* cytotoxicity against MDA-MB-231 breast cancer cells. This “green” combinatorial approach offers synergistic efficacy of both drugs loaded, and reduced side effects, being a promising biocompatible strategy for targeted breast cancer therapy. Wang et al. developed self-assembled nanomicelles of peptides derived from Lf through partial hydrolysis [213]. These nanomicelles presented a mean size of 50 nm, good stability, and demonstrated characteristics for CUR delivery. 

**Egg white proteins (EWPs)**. Recently, egg white proteins (EWPs) have been used to develop carriers for bioactive substances. EWPs-based delivery systems improve the bioactivities, bioavailability, and chemical stability of bioactive compounds. Liu et al. [214] highlighted that at present, the most widely used EWPs for nanocarrier development are ovalbumin and lysozyme, while ovotransferrin, ovomucin, and ovomucoid are less applied. 

**Spider silk and Worm silk Proteins.** Proteins derived from spider silk and silkworm silk have attracted much attention for bioactives’ delivery due to their excellent mechanical properties, biocompatibility, and biodegradability [215]. In the year 2022, Hu et al. [215] pointed out the antioxidant activity of silk sericin and its application as building blocks of bioactive materials for tissue engineering and drug delivery. Aghaz et al. developed pH-responsive silk sericin-based nanocarriers to co-deliver resveratrol and melatonin to MCF-7 breast cancer cells [216]. This smart pH-responsive nanoplatform demonstrated significant toxicity against breast cancer cells. Another research group [217] developed an antioxidant silk fibroin nanocarrier (with an average particle size of 40 to 105 nm) to deliver quercetin and trans-resveratrol.

**Ferritin** is another interesting protein used in nanoformulations [218]. Ferritin nanocages (consisting of 24 subunits arranged in the form of hollow structures with a diameter of 12 nm and an internal space of 8 nm) have been used to carry various molecules and ions [192]. Delivery vehicles based on a ferritin nanocage were used for the single encapsulation, co-encapsulation, and compartmentalized encapsulation of bioactive ingredients or drugs. These nanocarriers act as “multi-seated” vehicles for the encapsulation of different cargos simultaneously [219]. Safdarpour et al. developed ferritin nanoparticles loaded with *Glycyrrhiza glabra* saponin which attenuated the murine pneumococcal pneumonia [220].

(ii) ***NCs based on plant proteins***

Plants are safe, abundant, renewable, and cheap raw materials rich in bioactives for various applications in the pharmaceutical, medical, and food industries [192]. 

Since the possibility of transmitting the disease from plant sources to humans is rare, the plant proteins can be used as a replacement for animal proteins in nanotechnological purposes, avoiding, thus, the spread of animal diseases [192,221]. For this reason, the use of plant proteins to produce NCs for BCs has gained considerable interest in recent years. Plant protein-based NCs are able to control the release of their cargo over a long period of time [192]. 

**Zein Nanoparticles (ZeinNPs).** Zein, the main storage protein in maize (*Zea mays* L.), accounting for 30% to 60% of total protein content, readily self-assembles into various nanostructures, and it is a kind of excellent carrier material to construct nanocarriers for hydrophobic bioactives such as CUR [222]. ZeinNPs loaded with bioactive compounds have been used in the food industry [223], and also in the biomedical field [224]. The self-assembly formulation of Zein and dextran sulfate sodium (DSS) binary complex has been developed by the research group of Wang [224] for Que delivery. Moreover, DSS is a polyanionic derivative of dextran which possesses anti-inflammatory and antiviral activities. 

**Gliadin nanoparticles.** Gliadin is a type of protein of wheat gluten, with a molecular weight in the range of 25–100 kDa. Gliadins are soluble in alcohol and contain large amounts of the amino acid glutamine (about 40%) [192]. Depending on the pH, gliadin can self-assemble in different nanostructures, as a consequence of structural changes occurring in the gliadin tertiary structure [225].

Due to the presence of glutamine in its structure, gliadin forms many hydrogen bonds with the mucous layer and hence, gliadin nanoparticles (GNPs) have shown good potential in the preparation of oral formulations for the treatment of gastric diseases [192]. Moreover, glutamine residues play an important role in the antioxidant ability of gliadins and gliadin-based nanostructures [221]. 

GNPs were used to deliver many bioactive compounds (BCs) such as vitamin A, vitamin E, β-carotene, and flavonoids (e.g., RSV, CUR) [221]. Moreover, GNPs were loaded with vegetal extracts like grape skin extract which is rich in RSV [226], and its encapsulation within GNPs resulted in enhanced therapeutical properties.

Considering that gliadin is able to initiate an immune response in celiac disease patients, it is necessary to take this aspect into account when administering GNPs to these patients [221]. 

##### Peptide-Based Nanocarriers 

Interest in self-assembled peptide nanostructures (SPNs) increased in recent years [227]. Molecular self-assembly is a bottom-up process driven by noncovalent forces (such as hydrogen bonds, electrostatic interactions, van der Waals forces, π–π stacking, and hydrophobic interactions), resulting in highly organized and robust structures. Recently, self-assembled peptide development has been an effective measure to obtain new biomaterials that have been found in applications in materials science, drug delivery, regenerative medicine, and tissue engineering [228]. Amphiphilic peptides are considered ideal drug carriers because of their advantages: good biocompatibility and low toxicity to normal cells and tissues, design flexibility, functionality, and higher efficiency as novel drug-delivery carriers. SPNs can enhance drug release and cellular uptake, and are applied for the delivery of hydrophobic drugs (e.g., CUR), nucleic acid drugs, and peptide drugs [228]. 

Wang et al. [229] stated in their recent review that self-assembling peptides (SAPs) have enormous potential in biomedical applications, particularly in noninvasive tumor therapy. 

Kwon et al. [227] developed peptide vesicles (peptidesomes) with great potential in cancer therapy. The authors highlighted that peptidesomes are unique structures because peptides play dual roles as a self-assembly building block and as a bioactive functional unit. Pheophorbide *a* (Pa), a porphyrin derivative of chlorophyll *a*, has been loaded in peptidesomes and used as a photosensitizer for photodynamic therapy (PDT). The peptidesome←Pa showed tumoricidal photodynamic effects against squamous carcinoma 7 (SCC7) cells.

#### 5.2.2. Polysaccharides-Based DDS

Polysaccharides are another type of natural polymers used in DDS development, due to their hydrophilicity, biocompatibility, biodegradability, high availability, mucoadhesiveness, and they may additionally possess antimicrobial and anti-inflammatory properties [227,230,231]. Among the polysaccharides most often used in the development of DDS, the following can be mentioned: chitosan, alginate, starch, cellulose, hyaluronic acid, pectin, and dextran [232].

Some examples of DDS based on polysaccharides will be presented further.

##### Chitosan Nanoparticles and Chitosomes

Chitosan (CS) is a polysaccharide obtained by the deacetylation of chitin—a polysaccharide extracted from the extracellular matrix of marine crustaceans, crabs, shellfish, shrimp, and some fungi [233]. CS possesses many valuable properties such as biodegradability, biocompatibility, antibacterial, mucoadhesiveness, and hemostatic action, being applied in regenerative medicine and in the treatment of wounds, ulcers, and infections [233,234]. 

The loading of nanochitosan with bioactives results in nanoparticles with improved mucoadhesiveness and anticancer properties compared to the components. Thus, Chuah et al. [235] developed curcumin–chitosan nanoparticles with improved mucoadhesion and anticancer properties against human colorectal cancer cell line HT-29, compared to free CUR. The chemical interactions between the amine groups of CS nanoparticles and the keto groups of CUR is the key factor for loading the bioactive into nano-CS [236]. CS nanoparticles were successfully used to deliver simultaneously CUR and eugenol (EUG), proving to be powerful anticancer materials with a synergistic action against CaCo2 (colorectal adenocarcinoma) and MCF7 (breast adenocarcinoma) cells [236]. 

Soliman et al. loaded quercetin (Que) in CS NPs and obtained a nanoformulation with protective properties against cardiotoxicity, and also an improvement in the heart’s antioxidant defence system [141]. 

The use of a combination of CS with other biopolymers (polysaccharides or nucleic acids) gives rise to effective nanocarriers. Nalini et al. achieved CS–pectin nanoparticles that were used as Que nanocarriers [237]. A research group [238] developed polyplexes based on the combination of amphiphilic CS (based on the conjugation with polylactic acid) assembled with DNA, by using a solvent-free, simple, and low-cost method. These nanoformulations were loaded with β-carotene and presented high antioxidant activity.

Other CS-based nanocarriers are “chitosomes” which are chitosan-coated liposomes. The layer of CS on lipid vesicles increases the liposomes’ stability and can help improve the bioactives’ entrapment efficiency [239]. Chitosomes can be used for the transdermal delivery of bioactive compounds as summarized in a review [128]. 

##### Nano-Starch Particles (NSPs)

Starch is a natural polysaccharide made from two main polymers: *amylose* (a linear or slightly branched (1→4)-D-glucan) and *amylopectin* (a highly branched (1→4)-D-glucan) [240]. The main sources of starch are wheat, maize, potato, rice, beans, peas, tapioca, quinoa, and cassava. NSPs possess features such as biocompatibility, non-toxicity, and biodegradability that make them suitable for use as nanocarriers for bioactives. Thus, Dehghan-Baniani et al. [18] developed CUR-loaded starch micro/nanoparticles for treating osteosarcoma cancer. Jiang et al. [241] prepared NSPs from quinoa starch through the nano-precipitation method. These NSPs presented a high capacity for Que loading, prolonging Que bioactivity, and they have great potential as Que carriers. 

##### Nanocellulose

The cellulose nanocarriers are another attractive platform for bioactive delivery since they possess interesting features such as high flexibility to interact with other compounds, a high capacity for swelling/absorption of the liquid phase, high abundance in nature, large specific surface area, biocompatibility, biodegradability, good drug loading and binding capacities, good mechanical strength, stiffness, renewability, and low-cost [242,243]. Cellulose nanocrystals obtained from cotton fibers were used for CUR delivery for the healing of diabetics’ wounds [244].

Bacterial cellulose nanocrystals have been developed as a nanoplatform for the efficient delivery of phycocyanin, a bioactive produced by *Spirulina platensis*, which has antioxidant, antihyperalgesia, anti-inflammatory, antitumor, and anticancer properties. This nano-drug delivery system enhances the stability of phycocyanin in gastric pH conditions [245]. 

#### 5.2.3. DNA-Based DDS

Deoxyribo nucleic acid (DNA) is the “smartest” biomolecule that stores and conveys the genetic information in living organisms, and it is composed of two complementary strands linked through hydrogen bonding. These hydrogen bridges are responsible for the interesting properties of DNA and allow self-assembly with itself or with other biomolecules, giving rise to novel DNA materials with great potential for various applications [246] including biomedical ones such as DDS [247].

Starting from the 1980s when Seeman pioneered the DNA nanotechnology [248], many DNA nanostructures are obtained today. DNA is a programmable molecule and have sequence specificity, allowing the construction of DNA-based nanostructures through self-assembly [248], following the principle of complementary base pairing. In this way, valuable DNA nanocarriers can be obtained for bioactives’ delivery. Thus, Yuan et al. [249] developed DNA nanocarrier-loading curcumin (DNA-CUR) as a novel nanovector for effective CUR delivery enhancing its stability and bioaccessibility. Wang et al. [250] obtained DNA/chitosan nanoparticles for astaxanthin (ATX) delivery; these nanocarriers showed antioxidant capacity and enhanced cellular uptake efficiency. 

### 5.3. Drug Delivery with Living Cells and Cell Derivatives

The intrinsic stealth, biocompatibility, low immunogenicity, and targeting properties represent the main advantages of cell-derived biological vehicles [251]. 

Coating nanoparticles with natural cell membranes derived from red blood cells (RBCs), immune cells, stem cells, macrophages, platelets, and cancer cells is a promising method of biomimetic particle engineering [252]. These cell membrane-camouflaged NPs are biocompatible and inherit many of the properties of their source cells, and are used in targeted drug delivery. Wu et al. [253] developed a novel biomimetic DDS based on ursolic acid (UrA)-loaded nanoparticles (UrANPs) wrapped with a red blood cell membrane (RBCM). UrA is a natural pentacyclic triterpenoid with antitumoral properties. The obtained UrANPs-RBCM showed high stability, improved water solubility, and an anticancer effect by inducing the apoptosis and autophagy of non-small-cell lung cancer cells. RBCs or erythrocytes have attracted increasing attention in drug delivery applications since their first application as DDS in the 1970s [254], owing to their various characteristics such as biocompatibility, a life span of 120 days in blood circulation, high drug-loading capacity [255], enhanced targeting ability, and their excellent deformability [256]. Due to their interesting features, RBCs were applied especially in cancer therapy. Thus, for example, RBCs loaded with L-Asparaginase were used in the treatment of myeloid leukemia and pancreatic cancer [257]. 

Another type of NC was obtained by the entrapment in yeast cells of bioactives such as CUR, RSV, and chlorogenic acid [258]. The encapsulation of these BCs into yeast cells improved their stability. Yeast cells are promising delivery carriers owing to their ability to encapsulate both hydrophilic and hydrophobic bioactive compounds, biocompatibility, and biodegradability [259]. In addition, yeast-based delivery systems were applied to delivery peptides and small molecules, and for the oral delivery of insulin [260].

Another example of interesting biological cargo is based on diatoms (single-celled phytoplankton) nanoparticles (DNPs) which were applied for drug delivery in cancer therapy [261]. Diatoms possess interesting features: non-toxicity, high biocompatibility, low cost, porous structure, good permeability, great surface area, and easily modifiable surface chemistry, and that is why they are used as a scaffold for the development of drug delivery systems [262]. 

Plant cells have been also used as delivery vehicles, since they offer many advantages: high safety, low-cost, easy scale-up, and efficient bioencapsulation ability [260]. Thus, the plant cells of tobacco, lettuce, carrot, and potato were applied in the oral delivery of therapeutic proteins or antigens.

## 6. Other Types of Organic Nanocarriers

***Poly-ursolic acid nanoparticles (Poly-UrA NPs)***. UrA, a pentacyclic prevalent triterpenoid found in herbs, leaves, fruits, and blooms, with a wide variety of pharmacological properties (e.g., anti-diabetic, immunomodulatory, anti-inflammatory, hepatoprotective, or anticancer effects) has attracted increasing interests in the biomedical field. Hassanzadeh et al. [263] developed a platform based on poly-ursolic acid (poly-UrA) through a poly-condensation procedure and application of the nano-precipitation technique which enabled the self-assembly of poly-UrA into the NPs. The obtained Poly-UrA-NPs proved to be suitable nanocarriers for mithramycin-A, inducing synergistic toxicities towards the colorectal cancer cells. 

***Melanin nanocarriers***. Melanin (MEL), a natural pigment and a versatile biopolymer with many biological properties (antioxidant, anti-inflammatory, antitumor, and photoprotective against damaging by ultraviolet radiation), has been more exploited in the oncology field. Marcovici et al. reviewed the properties of melanins and the various applications of melanin nanoparticles (MEL-NPs) in the treatment of different cancer types [264]. They suggested a future direction related to the use of melanin as a carrier for natural anticancer compounds with hydrophobic character and reduced bioavailability. Liu et al. reported that MEL-NPs protected the heart against sepsis-induced myocardial injury via inhibiting ferroptosis and inflammation [265], which might be a novel nanostrategy to treat sepsis-induced myocardial injury. 

***Polydopamine nanocarriers***. Polydopamine (PDA) is considered a melanin mimetic material that imitates the composition and properties of the natural pigment. PDA was reported to have antineoplastic properties, selectively killing tumor cells without causing toxicity to healthy cells [264]. PDA nanostructures were applied as both therapeutic and diagnostic strategies predominantly in cancer management. PDA-NPs and derivatives were successfully applied for mucosal drug delivery [266]. Chen et al. [267] prepared spherical mesoporous polydopamine nanoparticles (mPDA-NPs) through an emulsion-induced interface polymerization method. CUR was loaded in these mPDA-NPs via π–π stacking and hydrogen bonding interaction. Mesopores disappeared after loading CUR. The obtained curcumin-loaded mesoporous polydopamine nanoparticles (CUR-mPDA NPs) were used for the prevention and treatment of radiation pneumonitis. 

***Shellac-based nanocarriers***. Shellac is a natural resin, an amphiphilic polyester secreted by the female lac beetle. This biopolymer has been recognized as safe by the Food and Drug Administration (FDA), and has applications in various fields including the pharmaceutical and food industries [223,268]. Yuan et al. developed shellac NPs for CUR delivery [206]. These nanocarriers presented physicochemical stability during *in vitro* simulated gastrointestinal digestion. 

## 7. Green Strategies for (Bio)organic Nanocarriers’ Design

### 7.1. “Green” Approaches to Design OBNs

Some “green” approaches to design (Bio)organic nanocarriers for bioactives’ delivery are presented further.

**Biomimetics/bioinspiration** is a new trend in modern *Green Nanotechnology*, giving rise to biocompatible and multifunctional nanocarriers. For example, liposomes that mimic biological membranes can be designed to encapsulate both hydrophilic and hydrophobic bioactive agents. Another example of bioinspiration is the mussel-inspired polydopamine (PDA) coating which is a convenient and effective approach to surface modification [269]. This strategy gives rise to effective nanocarriers for delivery bioactives like CUR [266].

**Self-assembly** is a “green” process based on the noncovalent interaction including hydrogen bonds, hydrophobic bonds, ionic bonds, π–π stacking, van der Waals, and electrostatic forces to the bottom-up design of functional architectures, such as DDS [270]. Moreover, self-assembly provides the self-healing of nanostructures such as in artificial cell membranes (liposomes). Three types of bio-entities containing BSA NPs and chlorophyll-labeled artificial cell membranes were “green” developed through the self-assembly strategy [271], providing interesting features for the development of novel multifunctional drug carriers. Supramolecular peptide assemblies were designed by employing the self-assembling properties of peptides and used as delivery vehicles [229].

**Ultrasound treatment** allows the size reduction of the particles, due to the shear forces/mechanical effects produced by the cavitation of microbubbles that form sound waves [240]. Santos et al. prepared polymeric NPs for RSV delivery, by using a sonication method and **Layer-by-Layer (LbL) self-assembly** [272]. LbL self-assembly consists in the successive steps of adsorption of oppositely charged polyelectrolytes upon a nanoparticle (NP) template containing a low-soluble drug, leading to onion-like multilayered nanostructures. The RSV nanoformulations obtained by Santos et al. showed good physical stability, homogenous particle size distributions, high encapsulation efficiency (>90%), and excellent cytocompatibility with Caco-2 cells (cell viability > 90%). 

The **emulsion-diffusion method** is another procedure to develop nanocarriers for bioactives. This method involves two steps: (1) the formation of an emulsion from a mixture of a partially miscible solvent in water containing the polymer, with an aqueous phase containing the stabilizer; (2) diffusion of the solvent from the internal phase by diluting the emulsion with large quantities of water, causing the entry of the polymer into a “non-solvent” phase resulting in polymer aggregates called “protonanoparticles” [273]. The **emulsion–diffusion** process was successfully used by Mohammed et al. for Que nano-encapsulation [274]. These nanoformulations were effective against MCF7 and CAL51 breast cancer cell lines. 

**Nanoprecipitation** is another “green” method to obtain nanoparticles (e.g., protein NPs, starch NPs, etc.). It is a simple and low-cost process with less danger of sample contamination [240]. Lam et al. [275] used the *Green Chemistry* principles to obtain BER-BSA NPs by using glucose instead of glutaraldehyde as a cross-linking agent for the modification of BSA. These NPs could be applied in a liver fibrosis treatment. BER is a phyto-bioactive, an isoquinoline alkaloid [276] with a wide range of biological activities including antioxidative, anticancer, neuroprotective, and anti-ischemic effects. 

**Figure 2:**
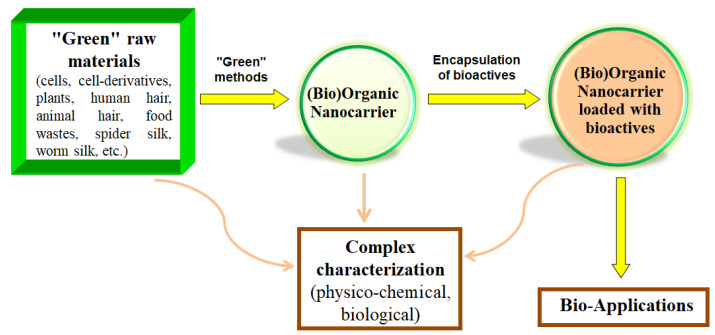
Schematic representation of “Green” development of (Bio)Organic Nanocarriers.

***Valorization of living systems and bio-derived wastes*.** “Green” strategies to design novel OBNs also include the valorization of living systems and bio-wastes and convert them into valuable nanomaterials (Figure 2). Thus, Bilge et al. [277] “green” synthesized NH_2_-MWCNTs from human hair wastes by the hydrothermal carbonization method, for biomedical applications. Other research groups produced carbon nanostructures through a “green” process from the onion vulgaris *(Allium cepa* L.) peels [278] and from tannin- polyphenols from *Mimosa* [279]. Food wastes (e.g., milk, cheese, eggs, animal proteins as collagen and gelatin sources, vegetables, fish, plants, hair wastes, chicken feathers, etc.) could be used as precursors for achieving valuable nanomaterials. For example, carbon nanocarriers can be *green* obtained from fruit wastes, tomatoes, *Gynostemma* plant, chitosan, red cabbage, green tea, and *Zingiberis* rhizome [280]. Other bioresources are living cells, marine organisms, spider silk, and worm silk (see Section 5). 

### 7.2. Specific Bio-Wastes and Living Systems for OBNs Development 

Modern technologies, particularly nanotechnology, present the opportunity to convert biodegradable waste into valuable products intended for human applications. The choice of bio-waste material (i.e., bio-derived residues) depends on the specific properties required for the nanocarrier and the intended application. Researchers often select materials based on their biocompatibility, biodegradability, and the ability to encapsulate and release therapeutic agents efficiently [281].

Several bio-waste materials can be used for the production of organic and biogenic nanocarriers with applications in drug delivery, targeted therapy, and other biomedical fields. Here are some specific bio-waste materials commonly explored for the production of organic and biogenic nanocarriers:

(i) Plant-derived waste
  - **Pectin from fruit peels**: Extracted from fruit peels, particularly citrus fruits, it can be utilized to form nanocarriers. Pectin has gelling properties and is biodegradable [282].
  - **Lignin from wood and plant residues**: a complex organic polymer found in plant cell walls and can be repurposed from wood and plant residues for nanocarrier development [283].
  - **Alginate from seaweed waste**: Extracted from seaweed waste and is commonly used for the production of nanocarriers. It is biocompatible and suitable for encapsulating various substances [284].
  - **Cellulose from plant residues**: extracted from plant residues, and can be modified to form nanocarriers with controlled release properties [285].
(ii) Animal-derived waste
  - **Chitosan from shellfish waste**: derived from the exoskeletons of shellfish, it is often used to create nanocarriers due to its biocompatibility, biodegradability, and mucoadhesive properties [286].
  - **Natural fibers**: hair wastes, chicken feathers, silk (produced by spiders and worm silk).
(iii) Waste derived from food industry and agriculture:
  - **Lipids** from edible oils, animal fats, and cooking oil waste originated from households, fast-food chains, restaurants, etc. can be a rich source of lipids for the preparation of lipid-based nanocarriers, such as liposomes or other lipid nanoparticles.
  - **Protein isolates from agro-industrial residues**: like leftover parts of crops, can provide protein isolates for developing protein-based nanocarriers [287].
  - **Gelatin from animal by-products**: obtained from animal by-products like bones and skin, they can be employed in the formulation of nanocarriers due to its film-forming and stabilizing properties [288].
  - **Whey Protein from dairy industry waste**: a by-product of the dairy industry, it can be utilized for creating nanocarriers with potential applications in drug delivery and the food industry [289].
(iv) ***Bioplastic waste***: Biodegradable polymers derived from bioplastic waste can be explored for the development of environment-friendly nanocarriers, nanofilms, etc. [290].
(v) ***Cell-derivatives*:** Cell membranes [291], bacterial ghosts, and yeast cell wall microcapsules [260] were used for the preparation of NCs.

Biodegradable waste, especially from fruits and vegetables after industrial processing, contains a wide range of bioactive compounds. These include flavonoids, phenols, tannins, steroids, triterpenoids, glycosides, anthocyanins, carotenoids, ellagitannins, vitamin C, and essential oils. This innovative utilization of biodegradable waste showcases the potential for a sustainable and eco-friendly approach to nanomaterial synthesis, contributing to both waste management and the production of valuable materials with beneficial properties [292].

## 8. Physico-Chemical and Biological Characterization of Bioactive Nanocarriers 

**Figure 3:**
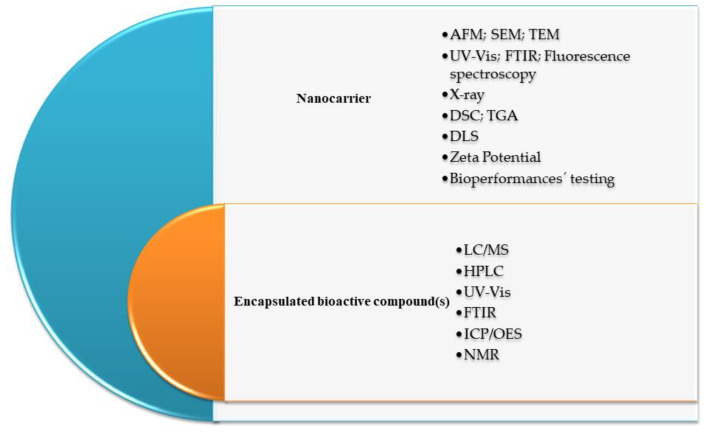
Characterization methods for nanocarriers loaded with bioactive compound(s).

The knowledge and control of basic physico-chemical properties of nanocarriers is one of the essential aspects in the development of safety products. In that sense, the first step is to characterize and determine the biological activity of the bioactive component itself, which should be incorporated into the appropriate carrier (Figure 3). 

The techniques most often used for the characterization of organic nanocarriers are the following [32,33,34,260,293]:

(i) *Microscopic techniques*: atomic force microscopy (AFM), scanning electron microscopy (SEM), and transmission electron microscopy (TEM), which offer relevant insight on the size, morphology, and surface texture;
(ii) *Spectroscopic techniques*: Ultra-violet visible (UV-Vis) absorption spectroscopy, Fourier-transform infrared spectroscopy (FTIR), fluorescence spectroscopy, dynamic light scattering [DLS, also known as photon correlation spectroscopy (PCS) or quasi-elastic light scattering (QELS)], laser diffraction (LD), zeta potential (ζ), X-ray diffraction (XRD), energy dispersive X-Ray spectrometry (EDS), small-angle X-Ray scattering (SAXS), small-angle neutron scattering (SANS), inductively coupled plasma–optical emission spectrometry (ICP-OES) which offer relevant insight on several physico-chemical properties such as the identification of spectral signatures of the components belonging to nanocarriers, understanding the interaction between components (bioactive compounds, matrix, etc.) of nano-DDS and between entire nano-DDS and the environment, particle size and particle size distribution, polydispersity index, particle charge, aggregation state, physical stability, structural organization and structural changes, degree of crystallinity, and elemental composition;
(iii) *Separation techniques*: High-performance liquid chromatography (HPLC) and liquid chromatography–mass spectrometry (LC-MS), used to separate, identify, and quantify various components, and also for the determination of the released drug content out of nano drug delivery systems, over time, in different physical conditions [294];
(iv) *Biochemical and biological techniques*: Offer relevant insight on antioxidant activity: the evaluation of the ability to scavenge short-life (by chemiluminescence technique) and long-life [by DPPH (2,2-diphenyl-1-picrylhydrazyl), and ABTS^●+^ (2,2-azinobis (3-ethylbenzothiazoline-6-sulphonic acid) method] free radicals; ferric reducing antioxidant power (FRAP), and ferrous ions (Fe^2+^) chelating activity (FIC) assays}, antimicrobial activity [agar well diffusion method; minimum inhibitory concentration (MIC) determination], cytotoxicity tests [using MTT (3-(4,5-dimethylthiazol-2-yl)-2,5-diphenyltetrazolium bromide) tetrazolium reduction assay], hemocompatibility evaluation [295,296];
(v) *Other techniques*: for example: thermogravimetric analysis (TGA), differential scanning calorimetry (DSC), provides information about thermal stability, purity, homogeneity, phase transitions of the sample, and the crystalline nature of the nanostructure [260].

The most commonly used methods for this purpose are UV-Vis method, HPLC, LC-MS, ICP-OES, FTIR, and the determination of antimicrobial activity and antioxidant activity using various tests such as DPPH, ABTS, FRAP, FIC [15,32,33,34,297,298]. After the incorporation of the bioactive compound into the carriers, the characterization of such systems is approached using other methods. In this sense, comprehensive characterization of nanocarriers relevant to their use and development is the analysis of droplet size and size distribution by the zeta sizer [299,300]. Another important part of characterization is the determination of the electrostatic surface potential [301]. Another relevant part of the characterization involves the morphological analysis of developed nanocarrier formulations [302,303]. 

The potential physical stability of nanoliposomes, nanoemulsions, or other nanocarrier systems, can often be predicted based on the size and surface charge of the droplets. Smaller and charged droplets have improved kinetic stability because they are more resistant to flocculation and sedimentation compared to the systems containing larger, non-charged droplets. By monitoring the distribution of droplet sizes in intravenous fat emulsions, it has been shown that parenteral nanoemulsions can contain droplets in a wide range of sizes: 100–400 nm, 700–1000 nm, and even 1000–3000 nm. The presence of such different populations of droplet sizes in the same formulation may be due to inefficient homogenization process or nanoemulsion instability [304]. In addition to droplet size, polydispersity index (PDI) is another critical parameter that describes the quality and homogeneity of nanocarrier systems [304]. PDI is a value from 0 (monodisperse distribution), 0.5 (relatively wide distribution), up to 1 [108]. Values less than 0.1 or 0.2 indicate a relatively narrow, unimodal distribution of droplet size and good quality of the nano-colloidal system, and better stability against destabilizing phenomena such as Ostwald’s maturation [305]. 

In the context of characterization and stability assessment of nanocarriers, optical light scattering techniques (PCS, LD) show certain limitations in terms of the inability to detect a small population of larger present droplets or the inability to detect the presence of other surfactant aggregates and the inability to distinguish spherical droplets from variable-shaped particles or crystals of a drug substance [306]. After dilution of the sample before measurement, the reversible destabilization phenomena such as flocculation or the appearance of larger aggregates may go unnoticed [183]. To overcome these limitations, the use of additional characterization methods such as nuclear magnetic resonance spectroscopy, infrared spectroscopy with Fourier transform [307], and microscopic analysis [308], is advisable. Optical light microscopy can be considered the simplest method for examining the structure of nanocarriers systems [308] but, this technique is limited because of the exact visualization of nanoparticles below 500 nm, and the inability to detect destabilizing phenomena such as coalescence and Ostwald maturation [309] or monitoring of phase changes. For accurate visualization of the nanostructure and determination of the droplet size, electron microscopy is becoming a necessary tool for nanosystems characterizing as a higher resolution technique [310]. Among electron microscopy techniques, transmission electron microscopy in combination with cryo-TEM is a method of choice for testing nanoemulsions in their original, native state, obtaining information on the size, shape of droplets, and the internal structure of the nanoemulsion system. It allows clear differentiation between nano-sized oil droplets and other structures possibly present in the nanoemulsion, such as vesicles, micelles, and liquid crystals [306]. In addition to these methods, rheological characterization is necessary and stability tests should determinate the stability of carriers as well as the bioactive compounds loaded. Due to the frequent high instability of nanocarrier systems, short-term and long-term stability tests are necessary. Long-term stability studies are usually performed by storing samples for a period, over 10 months, or 18–24 months, at temperatures ranging from 4 °C to 40 °C. 

After such a comprehensive analysis, it is very important to see how the active components are released from the carrier, which can be most easily seen by *in vitro* release tests. The methods used so far for this purpose, with more or less success, are sample-and-separate and continuous flow methods, *in situ* methods, and membrane barrier methods which include the side-by-side diffusion cell method and dialysis sac/bag method [311]. Among the currently available devices, the USP apparatus (flow cell) is recommended to characterize the *in vitro* release of the drug substance from nanocarriers, as well as the technique of reverse dialysis with the use of dialysis bags [138]. 

The biggest problem with nanocarrier systems is establishing and proving what is the effective encapsulated concentration of bioactive components into the carrier. It is also very difficult sometimes to prove how the active component itself is distributed along the carrier. Indirect methods are commonly used. For example, in the case of establishing the chlorophyll localization along the liposome membrane, it was monitored for fluorescence polarization and the fluorescence of chlorophyll quenching after insertion of the Que at the interface of lipid/water, in the vicinity of lipid polar heads and porphyrin macrocycle [136,137]. 

## 9. Fate of Bioactive-Nanocarriers Inside the Body

Once they have entered the body, in a physiological environment, NCs undergo *opsonization,* the process conducted by the body immune system, resulting in the formation of the protein corona enveloping the exogenous NCs. In this way, these newly achieved NCs will replace the original one thereby changing their physico-chemical and biological properties [312], lowering the efficacy of nano-drug delivery systems and their possibility to reach the target site [195]. Furthermore, through opsonization, macrophage removes the NCs from blood circulation. Therefore, the inhibition of nano-biointeractions between plasma proteins and entered NCs is a strategy to weaken the opsonization and delay the NCs clearance [312]. In their review, Owens and Peppas (2006) stated that another way to decrease the opsonization is a lower surface curvature of the nanomaterials. They also pointed out that the adsorption or grafting of poly(ethylene glycol) (PEG) to the NPs surface results in the creation of a hydrophilic protective layer around the NPs that is able to hinder the opsonin absorption via steric repulsion forces, thus blocking the opsonization process [313]. The experimental results obtained by Peng et al. [312] suggest that the formation of the NPs–albumin complex in advance is an effective strategy to inhibit the plasma proteins adsorption, since the preformed albumin corona around NPs serves as a protective coating for NPs. In this way, the opsonization process is weakened, reducing the NPs toxicity, and prolonging their blood circulation time. 

Adsorption, distribution, metabolism, and excretion (ADME), and toxicity profiles of nanocarriers are necessary to be determined for the safe application of organic nanocarriers [4]. Another concern is the fate of the bioactive which depends on the physicochemical and morphological properties and on the localization within the cellular environment. The surface chemistry of the nanocarrier is responsible for the nano-bio-interactions with cells, and determines the cellular uptake resulting in bioactive release inside the cell. 

## 10. Safety and Toxicity of Organic and Biogenic Nanocarriers

Great attention must be paid to the nanotoxicological aspects of OBNs, regarding the nano-biointeraction between nanocarriers and living systems. “Nanotoxicology” is a branch of nanomedicine related to the study of the toxicological effects of nanomaterials on the living systems (human body, animals, etc.) and environment. In their review, Umapathi et al. discuss the strategies and regulations adopted to mitigate the nanotoxicological problems [314]. They stated that the nanotoxicity is influenced by the physicochemical properties of nanomaterials, their synthesis methods, and routes of administration. Therefore, modulating their physicochemical properties and surface chemistry results in enhancing and controlling the efficiency and toxicity of nanomaterials. The safety of NCs for human health should be a topic for medicine, cosmetics, pharmacy, and the food industry. Nanomaterials can be completely digested and absorbed via the gastrointestinal tract, partially digested, or resistant to digestion from whereby the digestive system can pass into the bloodstream which can give some of the immunological reactions. Borel and Sabliov pointed out in their review [4] that the biocompatibility, biodegradability, and nanoparticle ADME profile are the key factors determining the safety of NCs. Another important issue is the toxicity of carbon-based materials which can be overcome by functionalization [87,88,91] with biomolecules or with artificial cell membranes [85,94]. Some studies [84,89] reported the role of mammalian peroxidases in the biodegradation of CNTs. These aspects were discussed in Section 4.

The small size of NPs facilitates their entry into biomembranes and other biological barriers, having a higher risk for the blood–brain barrier [315]. So, more tests are required to understand the degradation mechanism of NCs *in vivo* as well as their removal from the body. *In vivo* tests showed that nanoparticles around 40–70 nm can damage the liver, heart muscle and cause anemia. Nanoparticles can also enter the dermis more easily in places where the skin is thinner. The subsequent translocation of particles across the lymph nodes to the blood is also possible via transport to the lymph nodes by cutaneous macrophages. Even nanocarriers and nanoparticles can pose a great potential danger, the truth is that danger did not begin or end with the discovery of nanoparticles. It has always existed and applies to all chemical agents used as active principles. Among the different nanocarriers, lipid-based nanocarriers including nanoemulsions and liposomes were found to be safer [316] compared to metal nanoparticles which were found to be toxic [317]. However, the potential health risk of these nanomaterials is still not obvious. Additionally, nontoxicity depends on nanoparticle composition, dose, and size, so more studies are necessary to understand their consequences inside living cells [317]. 

The employment of “green” approaches to design eco-friendly nanoparticulate systems represents a necessity to overcome certain problems related to toxicity. 

## 11. Challenges and Opportunities in (Bio)organic Nanocarriers Loaded with Bioactives

### 11.1. Development of Novel “Green” Strategies

The development of novel “green” or sustainable strategies to improve the bioavailability and the stability of bio-nanocarriers is a current challenge. For example, the deep penetration of solid tumors is a real challenge. Thus, ***bionicstrategies*** were developed for the surface modification of OBNs in order to achieve the deep penetration of tumors. In a recent review [318], the authors highlighted two bionic ways for surface modification: (1) mimicking some molecules on the surface of living systems to promote penetration; (2) applying the whole outer layer of the living entities like cell membranes or virus shell on nanocarriers’ surface. 

***Co-delivery.*** The preparation of multifunctional nanocarriers by coencapsulating bioactive molecules enables synergistic therapies, and is challenging due to the different physicochemical properties of the cargo molecules [319]. An opportunity to design effective nanocarriers for cancer treatment is based on the synergistic action of the combination of natural active ingredients and chemotherapy drugs. So, natural bioactives such as betulinic acid (BA), Que, and CUR decrease the side effects of chemotherapy drugs (e.g., PTX), and enhance their cytotoxicity against cancer cells [41]. The research group of Ott [320] developed squalene (Sq)-based NLCs co-encapsulating two actives—one antitumor drug, pemetrexed (Pm), and one bio-flavonoid, hesperidin (Hes). Sq was isolated from amaranth seeds. The system Sq-NLC-Pm-Hes showed excellent antioxidant activity which was greater than each component alone. 

There are two main strategies for the co-encapsulation of natural active ingredients and chemotherapy drugs: (i) a carrier-linked prodrug delivery system (through chemical bonding at the same functional group as ester or disulfide), self-assembled into nanoparticles, and (ii) physical encapsulation in nanocarriers [41]. 

***Combining therapeutical strategies.*** Combination therapy provides various advantages such as synergistic effects, enhanced stability, solubility, and cell uptake of bioactive agents, and also reducing drug-related toxicity. For example, SWCNTs loaded with CUR serve not only as scaffolds, but also as thermal ablation agents, enhancing the antitumor activity of CUR [92]. Due to the high optical absorbance at 808 nm, SWCNTs can act as a photothermal therapy agent for “tumor cooking”, due to the excessive local heating upon laser illumination. Meng et al. [321] developed bifunctional polydopamine-modified curcumin-loaded silk fibroin composite nanofibrous scaffolds which were used as a localized drug delivery system in combination therapy (photothermal therapy with chemotherapy) in osteosarcoma treatment.

***Oral delivery*** is an actual tendency, due to its multiple advantages including sustained and controlled drug delivery, but the design of oral nanocarriers is a challenge, considering that they must be formulated to withstand the pH of gastric fluid, and the action of digestive enzymes from the gastrointestinal tract (GIT), and to prevent irritation after oral applications. A recent review pointed out that chitosan nanoparticles (CS-NPs) possess many advantages for the oral delivery of phytochemicals (e.g., CUR, Que, RSV, BER, ATX, lutein, etc.) such as excellent stability in the gastrointestinal environment, excellent mucoadhesiveness, non-toxicity, biodegradability, and biocompatibility [322]. Milović et al. [323] reported the first demonstration of the self-emulsifying phospholipid suspension approach combined with diatom particles as solid drug-carriers for oral formulations of hydrophobic drugs. This novel approach is low-cost, and provides enhancement of the dissolution rate of poor water soluble drugs, improving their therapeutic efficacy. 

***Biomimetic and bioinspired strategies*** in drug delivery are in the spotlight of scientists and have gained considerable interest in the last years. Thus, the biomimetic membranes (liposomes) were used to enhance the oral bioavailability of macromolecular drugs [324]. In their review, Sabu et al. [325] pointed out that targeting the cell can be enhanced by mimicking the texture and shape of the cell. Moreover, the **bioinspired and biomimetic nanocarrier** systems impart biocompatibility and less/no toxicity during drug delivery applications. The authors also highlighted the roles of surface chemistry, and shape in modulating NCs’ interactions with cells. 

Increasing the capacity of ***targeting the delivery*** of drugs to tumors is another challenge. The surface of nanocarriers can be modified with active targeting molecules like folic acid or hyaluronic acid to target cancer cells [41].

***The use of living systems and bio-wastes*** is another choice in the *smart delivery*/*targeted delivery* of bioactives. Thus, using **cell-derived biomimetic nanocarriers** is a strategy to overcome many drawbacks of traditional NCs [326], providing a more effective strategy for cancer therapy. Cell-based NCs face many challenges, since they have the advantage of ‘mimicking nature’, and enhanced bioperformances such as low toxicity, ability to pass through biological barriers, long-term circulation in the body, and improved interactions with other cells, thus protecting the bioactive carried. 

### 11.2. Limitations and Future Strategies in the Development of OBNs

Despite the OBNs advantages, researchers face several challenges in developing OBNs as successful materials [327]. Transitioning from small-scale laboratory processes to large-scale industrial applications requires a re-evaluation of factors such as variable process parameters, material optimization, and the quality of biogenic materials. 

In comparison to synthetic NP preparation, controlling the physicochemical properties of biogenic NPs, including size and shape uniformity, stability, surface functionalization, and analysis, presents critical challenges [328,329]. Critical issues in synthesizing large-scale biogenic NPs involve identifying appropriate biomolecules, preventing bio-waste degradation before implementation, and addressing concerns related to storage and collection. Parameters such as temperature, pH, and oxygen levels must be meticulously monitored and controlled to ensure consistent and efficient NP production. Consequently, scalable and innovative methods maintaining efficiency and quality become crucial [283].

Biogenic NPs are still under investigation for applications in wearable technology, presenting an avenue for developing a secure process with fewer hazardous effects [330]. Extracts from aquacultural and horticultural food waste serve as excellent sources of bioactive compounds, making them ideal for nanoparticle synthesis with potential applications in the pharmaceutical and biomedical fields [331]. 

The use of organic and biogenic nanocarriers, although promising, is not without limitations. Several challenges and constraints need to be addressed for the effective application of these nanocarriers. Here are some limitations:

- **Scale-up challenges**: Translating laboratory-scale production to large-scale industrial processes poses challenges in maintaining reproducibility, consistency, and cost-effectiveness [292,332].
- **Biocompatibility and toxicity concerns**: Some organic and biogenic materials may raise concerns related to biocompatibility and potential toxicity, necessitating rigorous testing and evaluation before clinical applications [333].
- **Control over physicochemical properties**: Achieving precise control over the physicochemical properties of organic and biogenic nanocarriers, such as size, shape, and surface charge, can be challenging compared to synthetic counterparts [334].
- **Stability issues**: Stability of nanocarriers during storage and transportation is a critical concern. Aggregation, degradation, or changes in properties over time can impact their effectiveness [9,335].
- **Limited loading capacity**: Some organic materials may have limited capacity to encapsulate or carry therapeutic payloads, restricting the amount of drug or bioactive substance that can be delivered [336].
- **Biodegradation variability**: The biodegradation rates of organic and biogenic nanocarriers may vary, leading to unpredictable release profiles of encapsulated substances [337].
- **Complexity in formulation**: Formulating organic and biogenic nanocarriers can be complex due to the variability of natural materials, making it challenging to achieve a consistent performance [338,339].
- **Low drug loading efficiency**: Achieving a high drug loading efficiency in organic and biogenic nanocarriers may be challenging, leading to suboptimal drug delivery efficacy [339].
- **Limited targeting efficiency**: Achieving targeted delivery to specific tissues or cells may be less efficient compared to synthetic nanocarriers, impacting the therapeutic efficacy [339].
- **Regulatory challenges**: The regulatory approval process for organic and biogenic nanocarriers can be more intricate due to the inherent variability of natural materials, requiring comprehensive safety and efficacy assessments [340,341].
- **Lack of standardization**: The lack of standardized methods for the production and characterization of organic and biogenic nanocarriers hinders their widespread adoption and comparison between studies [342].

## 12. Concluding Remarks and Future Directions 

The nanoencapsulation of bioactive compounds in nanocarriers is known as a field of nanotechnology whose purposes are the entrapment and delivery of bioactive principles, controlling their release, increasing their bioavailability, and increasing their stability. The era of nanomaterials has begun. The science of nanomaterials is advancing day by day and will continue to advance in terms of finding better test methods for the safer handling and use of nanoproducts based on bioactive compounds incorporated in nanocarriers. 

Various methods are available for encapsulating bioactives, and the application of (bio)organic nanocarriers is considered as a new and promising technique. Having in mind the structure and nature of the bioactive component(s), it is possible to make an adequate selection of nanocarriers (liposomes, micelles, polymer nanoparticles, emulsions, etc.) and thus affect the distribution, stability, and effectiveness not only of the active component(s) but also the quality of the final product.

Multi-functionality is another aspect of great interest, which must be taken into account when preparing a nanocarrier. A nanotransporter can be designed in such a way as to perform several functions (antioxidant, antimicrobial, antitumoral, and anti-inflammatory activities, etc.). A particular shape and surface chemistry for organic nanocarriers that enable biocompatibility and endosomal escape assures a better delivery of the bioactive to the target.

The use of natural raw materials for developing valuable nanocarriers for bioactives must be in the spotlight of scientists since they are “green” materials that offer many advantages such as eco-friendliness, biodegradability, and biological activities. Moreover, “green” strategies such as biomimetics, bioinspiration, and bionics must be considered in order to achieve advanced nanocarriers. 

Biomolecules (e.g., peptides, proteins, nucleic acids, etc.), cells, cell-derived wastes, and other living systems can be used as building blocks to bio-design novel nanocarriers that are biocompatible, biodegradable, and low/non-toxic. Moreover, the integration of biological entities into nanocarriers’ formulations will bring impressive new bioactivities (like biocompatibility, low toxicity, antioxidant properties, antimicrobial, anti-inflammatory, and anticancer activities). 

The use of natural bioactives as encapsulating agents in nanocarriers is another trend in *Green Nanotechnology*, considering their properties: safety, biocompatibility, biodegradability, and bioactivities. The phyto-bioactives are the most attractive in various therapies since they are derived from plants that are highly abundant in nature, and possess many biological activities (e.g., antioxidant, antimicrobial, antiproliferative, anti-inflammatory properties).

The valorization of bio-wastes from the food industry, agronomy, hair salons, animal wastes, silk fibers produced by spiders and worms, vegetal wastes, and so on, is an important and low-cost issue for the development of safe and performant organic nanocarriers. 

The commercial aspect of bioactive carriers involves considerations related to their production, market demand, applications, and potential economic benefits. Certain categories of OBNs have already been commercialized in the field of cosmetics (e.g., creams, lotions), medicine (vaccines, drug delivery systems, phytosomes, food supplements), and the food industry (nanocarriers for nutraceuticals).

The field of nanotechnology has shown great promise in drug delivery, offering the potential for more targeted and efficient therapeutic interventions. However, several challenges remain, and future progress is needed to fully harness the capabilities of nanotechnology in drug delivery (targeting specific cells or tissues, biocompatibility and safety, stability and shelf life, scalability of production, drug loading and release kinetics, understanding biodistribution and pharmacokinetics, overcoming biological barriers, personalized medicine approaches and regulatory approval, and standardization). Advancements in these areas will contribute to the development of more effective and clinically viable nanotechnology-based drug delivery systems. 

OBNs have indeed made a revolutionary impact on the nanotechnology and biomedical fields by addressing challenges in drug delivery, diagnostics, bio-sensing, and regenerative medicine. Their unique properties have paved the way for more targeted and effective therapies, as well as advanced applications in imaging and diagnostics. It is important to note that ongoing research and development in this field may lead to further innovations and applications. 

## Figures and Tables

**Table 1:** Some plant bioactive compounds and the nanocarriers transporting them.

Natural Bioactive Compound	Nanocarrier	Source	Ref.
Naringenin	Polymeric nanoparticles, micelles, liposomes, solid lipid nanoparticles (SLNs), nanostructured lipid carriers (NLCs), nanosuspensions, nanoemulsions	Fruits, tomato, cherries, cocoa	[25,35]
Honokiol	Nanoliposomes (nanosomes)	Magnolia	[22]
Resveratrol	Liposomes, polymeric nanoparticles, SLNs, lipospheres, dendrimers	Red grapes, peanuts	[21,36]
Glycyrrhetinic acid	Micelles, liposomes	Licorice root	[25,37]
Rutin	BSA nanoparticles	Plants, *Ruta graveolens*	[38]
Curcumin	Micelles, liposomes nanogels, nanodiscs	*Curcuma longa* species Turmeric extract.	[39,40,41,42]
Quercetin	Micelles, liposomes	Plants, guava leaves, *Lagerstroemia speciosa*, grapes	[41]
Luteolin	Nanoemulsions, liposomes, SLNs, dendrimers	Herbs, fruits and vegetables	[43]
Carotene	Nanoemulsions, liposomes, SLNs, dendrimers	Carrots, tomatoes	[44]
α-Tocopherol	Nanoemulsions	Herbs, herbal mineral oils	[45]

**Table 2:** Some bioactive compounds encapsulated within polymer nanocarriers.

Encapsulated Extract/Compound	Nanocarrier	Field of Use	Activity	Ref.
Essential oils (geranium, *Geranium maculatum*, and bergamot, *Citrus bergamia*)	Polyethylene glycol (PEG) NPs; Chitosan NPs	Cosmetics	Repellent	[64,65]
Curcumin extract	Polymer NPs	Food, pharmaceutical, and cosmetic use	Coloring and antioxidant agent, food additive, anticancer	[66,67]
Catechins from white tea extract	Polymer NPs	Food	Antioxidant	[68]
Oregano essential oil	Chitosan NPs	Pharmacy	Antioxidant and antifungal	[69]
*Cuscuta chinensis* seed extract	Polymer NPs	Medicine	Hepatotoxicity	[70]
*Ziziphus jujuba* extract	Chitosan NPs	Pharmacy	Antioxidant	[71]
*Ginkgo biloba* extract	PELGE NPs	Medicine	Antioxidant	[72]
Extract of neem (*Azadirachta indica*)	Polymeric nanofibers	Biomedicine	Wound dressing, transdermal carriers	[73]
Zoledronic acid	Polymer NPs	Medicine	Cancer therapy	[74]
Kaempferol	PEG 1000 succinate nanosuspensions	Medicine	Antitumor activity	[75]

**Table 3:** Examples of some bioactives encapsulated within lipid nanocarriers.

Encapsulated Bioactive Compound/Extract	Nanocarrier	Field of Use	Activity	Reference
*Spinacia oleracea* L. extract	Liposomes	Cosmetics	Antioxidant	[108]
*M. communis* extract	Liposomes	Food preservatives	Antioxidant and antimicrobial activity	[109]
*Oryza sativa* L. extract	Niosomes	Cosmetics	Anti-age	[110]
Saffron extract	Liposomes	Medicine, food, textiles	Food additive; memory enhancing; anticonvulsant, antidepressant, antioxidant, antitumor	[111]
Omega-3 fatty acids	Liposomes	Food	Nutritional quality	[112]
Phloridzin	Liposomes	Food industry, pharmaceutics	Functional food and pharmaceutical applications	[113]
Curcumin	Nanoemulsions	Food industry	Antioxidant	[114]
Polymethoxyflavones	Nanoemulsions	Pharmaceutics	Anticancer	[115]
*Opuntia oligacantha* extract	Nanoemulsions	Food industry	Improving the postharvest life of food	[116]
*O. stamineus* ethanolic extract	Nanoliposomes	Cosmetics	Antioxidant and antimicrobial effect	[117]
*M. communis* extract	Nanoliposomes	Pharmacy	Treatment of parasitic and antimicrobial diseases	[118]
*B. vulgaris* and *B. integerrima* extract	Liposomes	Food	Antioxidant activity	[119]
*Hibiscus sabdariffa* L. flower extract	Liposomes	Food	Antioxidant	[120]
*Phyllanthus niruri* extract	Nanoemulsions	Cosmetics	Antibacterial activity	[121]
Insulin	Nanoliposomes	Medicine	Regulate blood glucose levels	[122]
Elemene	Liposomes, SLNs	Medicine	Cancer therapy	[123]
Albumin	Liposomes	Medicine	Hepatic fibrosis therapy, ovarian, breast, and colon therapy	[124] [125]
Omega-3 fatty acid and quercetin	NLCs	Cosmetics	Antioxidant and photoprotective activities	[126]

## Data Availability

The data were included in the text.

## Abbreviations

ACE	Angiotensin I-converting enzyme
ADME	Adsorption, distribution, metabolism, and excretion
ATX	Astaxanthin
BA	Betulinic acid
BCs	Bioactive compounds
BEO	Bergamot essential oil
BER	Berberine
BILs	Bilosomes
BNCs	Biogenic nanocarriers
BSA	Bovine serum albumin
CBNs	Carbon-based nanomaterials
CaCo2	Colorectal adenocarcinoma cells
Chl	Chlorophyll
CMC	Carboxy methyl cellulose
CNOs	Carbon nano-onions
CNTs	Carbon nanotubes
CNT-RSV	Carbon nanotubes loaded with resveratrol
CQDs	Carbon quantum dots
CS	Chitosan
CS-NPs	Chitosan nanoparticles
CUR	Curcumin
CUR-mPDA NPs	Curcumin-loaded mesoporous polydopamine nanoparticles
DDS	Drug delivery system
DLPLG	Poly (DL-lactide-co-glycolide)
DNA	Deoxyribonucleic acid
DNPs	Diatom nanoparticles
DSS	Dextran sulfate sodium
DWCNTs	Double walled carbon nanotubes
EPO	Eosinophil peroxidase
EUG	Eugenol
EVs	Extracellular vesicles
EWPs	Egg white proteins
FDA	US Food and Drug Administration
FNPs	Fibrinogen nanoparticles
GA	Gallic acid
GIT	Gastrointestinal tract
GNPs	Gliadin nanoparticles
HepG2	Human liver carcinoma cell
Hes	Hesperidin
HNK	Honokiol
HPTX NPs	Disulfide bond bridged paclitaxel-pentadecanoic acid conjugate
HSA	Human serum albumin
HT-29	Human colorectal cancer cell line
L929	Mouse fibroblast healthy cells
α-Lac	α-Lactalbumin
(LbL)	Layer-by-Layer
Lf	Lactoferrin
LNCs	Lipid-based nanocarriers
LT4	Levothyroxine
LUT	Luteolin
LYC	Lycopene
MCF7	Human breast cancer cell line
MEL	Melanin
MEL-NPs	Melanin nanoparticles
mPDA-NPs	Mesoporous polydopamine nanoparticles
MPO	Myeloperoxidase
MWCNTs	Multi- walled carbon nanotubes
Nab-technology	Nanoparticle albumin-bound technology
NRG	Naringenin
NC	Nanocarrier
NDs	Nanodiamonds
NLCs	Nanostructured lipid carriers
NP	Nanoparticle
NSPs	Nano-starch particles
NTs	Nanotubes
OBNs	Organic and biogenic nanocarriers
ONs	Oleander (*Nerium oleander*) niosomes
OC	Ovarian cancer
OVA	Ovalbumin
PAMAM	Poly(amidoamine)
PC3	Prostate cancer cell line
PDA	Polydopamine
PDA-NPs	Polydopamine nanoparticles
PDT	Photodynamic therapy
PEG	Polyethylene glycol
PEI	Polyethylenimine
PELGE	Copolymers (monomethyl poly(ethylene glycol)–poly (lactide-co-glycolide)–monomethyl-poly(ethylene-glycol)
PGA	Polyglycolide
Pa	Pheophorbide *a*
PLA	Polylactide
PLGA	Copolymer poly (lactide-co-glycolide)
Pm	Pemetrexed
poly-UrA	Poly-ursolic acid
poly-UrA NPs	Poly-ursolic acid nanoparticles
PPy	Polypyrrole
PTX	Paclitaxel
QS	Quatsomes
QS-UA	Quatsomes loaded with usnic acid
Que	Quercetin
RBCs	Red blood cells
RBCM	Red blood cell membrane
RSA	Rat serum albumin
RSV	Resveratrol
SAPs	Self-assembling peptides
SCC7	Squamous cell carcinoma 7 cells
SLNs	Solid lipid nanoparticles
SPNs	Self-assembled peptide nanostructures
Sq	Squalene
SUVs	Small unilamellar lipid vesicles
SYL	Silymarin
SWCNTs	Single-walled carbon nanotubes
TEBVs	Tissue-engineered blood vessels
TF	Transferrin
TPs	Terpesomes
TPGS	d-alpha-tocopheryl polyethylene glycol 1000 succinate
UrA	Ursolic acid
UA	Usnic acid
ZeinNPs	Zein Nanoparticles
